# Glucolipotoxicity initiates pancreatic *β*-cell death through TNFR5/CD40-mediated STAT1 and NF-*κ*B activation

**DOI:** 10.1038/cddis.2016.203

**Published:** 2016-08-11

**Authors:** Marta Bagnati, Babatunji W Ogunkolade, Catriona Marshall, Carmen Tucci, Katie Hanna, Tania A Jones, Marco Bugliani, Belinda Nedjai, Paul W Caton, Julius Kieswich, Muhammed M Yaqoob, Graham R Ball, Piero Marchetti, Graham A Hitman, Mark D Turner

**Affiliations:** 1 Barts and The London School of Medicine and Dentistry, Queen Mary University of London, London, UK; 2 Interdisciplinary Biomedical Research Centre, School of Science and Technology, Nottingham Trent University, Nottingham, UK; 3 Department of Clinical and Experimental Medicine, University of Pisa, Pisa, Italy; 4 Leukocyte Biology Section, National Heart and Lung Institute, Imperial College, London, UK; 5 Diabetes and Nutritional Sciences Division, School of Medicine, King's College London, London, UK

## Abstract

Type 2 diabetes is a chronic metabolic disorder, where failure to maintain normal glucose homoeostasis is associated with, and exacerbated by, obesity and the concomitant-elevated free fatty acid concentrations typically found in these patients. Hyperglycaemia and hyperlipidaemia together contribute to a decline in insulin-producing *β*-cell mass through activation of the transcription factors nuclear factor kappa-light-chain-enhancer of activated B cells (NF-*κ*B) and signal transducer and activator of transcription (STAT)-1. There are however a large number of molecules potentially able to modulate NF-*κ*B and STAT1 activity, and the mechanism(s) by which glucolipotoxicity initially induces NF-*κ*B and STAT1 activation is currently poorly defined. Using high-density microarray analysis of the *β*-cell transcritptome, we have identified those genes and proteins most sensitive to glucose and fatty acid environment. Our data show that of those potentially able to activate STAT1 or NF-*κ*B pathways, tumour necrosis factor receptor (TNFR)-5 is the most highly upregulated by glucolipotoxicity. Importantly, our data also show that the physiological ligand for TNFR5, CD40L, elicits NF-*κ*B activity in *β*-cells, whereas selective knockdown of TNFR5 ameliorates glucolipotoxic induction of STAT1 expression and NF-*κ*B activity. This data indicate for the first time that TNFR5 signalling has a major role in triggering glucolipotoxic islet cell death.

In 2011, it was estimated that there were 347 million people worldwide living with diabetes.^1^ Moreover, the incidence of diabetes continues to grow at an alarming rate, with the figure in 2030 projected to be more than double that reported in 2000.^2^ There are a limited number of options to treat type 2 diabetes (T2D), and furthermore oral and injectable medications often become less effective over time. Thus, there is an urgent need to better understand the causes of diabetes, and to identify new targets for the development of novel treatment strategies.

Hyperglycaemia and hyperlipidaemia together contribute to the gradual loss of *β*-cell function that has been observed in patients with T2D. In addition to the failure of compensatory hypersecretion to overcome insulin resistance, reduction in *β*-cell mass through increased apoptosis is a key component of T2D.^3, 4^ Increased metabolic stress results in the activation of the transcription factors nuclear factor kappa-light-chain-enhancer of activated B cells (NF-*κ*B)^5, 6^ and signal transducer and activator of transcription (STAT)-1.^7^ These then trigger the synthesis of a number of *β*-cell proteins, including the cytokine interleukin (IL)-1*β*.^8^ This is then released from the cell, whereupon it is able to bind the *β*-cell surface IL-1 receptor and amplify both NF-*κ*B activation and the subsequent cytokine response that is driven through activation of nucleotide-binding oligomerization domain receptor family, pyrin domain containing 3 inflammasomes.^5^ In addition, inflammasome activation can also lead to caspase-1-mediated cell death through a process termed pyroptosis, which both fragments DNA and leads to pore formation in the plasma membrane.^9^

As well as these IL-1*β*-mediated cytokine response pathways, a number of chemokines are also synthesised and released by *β*-cells following glucolipotoxic activation of NF-*κ*B.^5^ These serve to attract circulating immune cells, which then infiltrate the pancreas and are specifically drawn towards *β*-cells.^10, 11^ Here these immune cells release their own cocktail of cytokines. Along with glucolipotoxicity-induced *β*-cell IL-1*β* release, this localised toxic milieu effectively destroys *β*-cells.

Although we know many of the events resulting from NF-*κ*B activation, the initial trigger of glucolipotoxic NF-*κ*B activation remains poorly defined. Endoplasmic reticulum (ER) stress is one mechanism that has been proposed to initiate this response.^12^ However, as ER stress is typically induced by an increase in the synthesis of client proteins, this is more likely to occur in response to increased transcriptional activity and the resulting increase in protein synthesis and ER throughput. Hence, ER stress is more likely to be a consequence rather than cause of the increased transcriptional activity and/or islet inflammation. There are some exceptions, such as Wolfram syndrome where there is a coding mutation in the protein wolframin that is thought to regulate ER Ca^2+^ flux and thereby ensure protein fidelity and the degradation of misfolded proteins.^13^ However, as this and similar genetic disorders are relatively rare they are unlikely to contribute to ER stress in the majority of people with T2D.

Oxidative stress is another mechanism commonly championed, as the initial driver of gluco-or lipotoxic NF-*κ*B activation in *β*-cells. This is also questionable, however, since reactive oxygen species (ROS) trigger a rapid spike in intracellular Ca^2+^ that results in transient exocytosis of insulin.^14, 15^ Indeed, there is increasing evidence to suggest that ROS are essential for normal *β*-cell function, including insulin secretion.^16^ Furthermore, some anti-oxidants potently inhibit glucose-stimulated insulin secretion, not enhance it.^17^ This may help explain why levels of the H_2_O_2_-inactivating enzymes glutathione peroxidase and catalase are particularly low in *β*-cells, when compared, for example, with expression level in the liver.^18^ This does not mean that *β*-cells are incapable of sustaining damage from excessive oxidative stress. However, given the routine high glycolytic throughput of *β*-cells and the general tolerance of *β*-cells against levels of ROS that would be damaging to other cells, it would seem incongruous were glucolipotoxicity to exert a disproportionately damaging effect upon pancreatic function.

To more accurately define the initial mechanisms triggering glucolipotoxicity-induced *β*-cell dysfunction, we used a combination of microarray expression profiling, independent quantitative PCR analysis, western blotting, confocal microscopy, and NF-*κ*B activity assays to identify those genes and proteins, whose expression is most sensitive to glucose and fatty acid environment. Our data indicate that of the genes most likely to be associated with cell death pathways, tumour necrosis factor receptor (TNFR)-5 (also known as CD40) is the most highly upregulated and is able to induce STAT1 and NF-*κ*B activation. Importantly, this pattern of upregulation was not restricted to rat insulinoma cells alone, but also isolated mouse islets from high-fat-fed mice and human islets, indicating that this may represent a conserved cell death activation mechanism.

## Results

**Figure 1:**
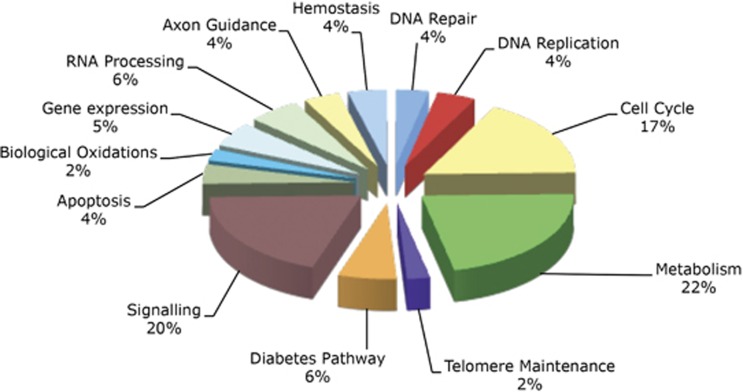
Effect of glucolipotoxicity on *β*-cell ontology. Comparative analysis of gene expression from INS-1 cells incubated for 72 h in RPMI-1640 media supplemented with or without 28 mM glucose, plus 200 *μ*M oleic acid, and 200 *μ*M palmitic acid. Data are compiled from six independent microarray analyses (three per experimental group). Reactome pathway analysis (http://www.reactome.org) identified 13 significant biological functions/pathways (*P*<0.05)

To better understand the mechanisms by which chronic exposure to high glucose and fatty acids alters cellular function, we incubated INS-1 rat pancreatic *β*-cell insulinoma cells for 72 h in Roswell Park Memorial Institute (RPMI)-1640 media and media supplemented with 28 mM glucose, 200 *μ*M oleic acid, and 200 *μ*M palmitic acid. Total RNA was isolated and hybridised to Affymetrix rat microarray chips (Santa Clara, CA, USA). Approximately 10% of INS-1 transcripts underwent a twofold or greater change in expression that was statistically significant (Supplementary Appendix Table 1). This included genes linked to apoptosis and related signalling pathways, biological oxidation, nucleic acid processing, and repair (Figure 1). To better understand how altered function in these genes correlates with human disease, we employed Metacore analysis algorithms (https://portal.genego.com) to identify the 25 diseases most closely associated with our data set. Table 1 indicates the degree of significance, along with enrichment expressed as the ratio of the number of genes that would be expected to be associated to each specific disease by chance (shown in green) compared with the number of the differentially expressed genes enriched in our data set (shown in red).

For molecules linked to inflammation and apoptosis, we found changed expression in a number of tumour necrosis receptor (TNFR) superfamily members and associated factors. Of these *TNFRSF5*, the gene encoding TNFR5 was found to be the most highly upregulated with 2.23-fold expression (*P*<0.01; Table 2). To validate these results, we performed independent quantitative real-time PCR (qRT-PCR) analysis of *TNFRSF5*, as well as the genes encoding TNFR1 and TNFR6 (also known as Fas) that have previously been shown to mediate cytokine-induced islet inflammation and apoptosis. Again, we observed significant increase in *TNFRSF5* expression (3.62-fold; *P*<0.01) induced by high glucose and fatty acid environment (Table 2). *TNFRSF1A* was also upregulated by greater than twofold, but variable response meant that this was not deemed statistically significant in the samples examined. *TNFRSF6* by contrast was only modestly upregulated, with this not found to be statistically significant in the PCR experiments. As a consequence, we focused our investigation on TNFR5 for the remainder of this study.

**Figure 2:**
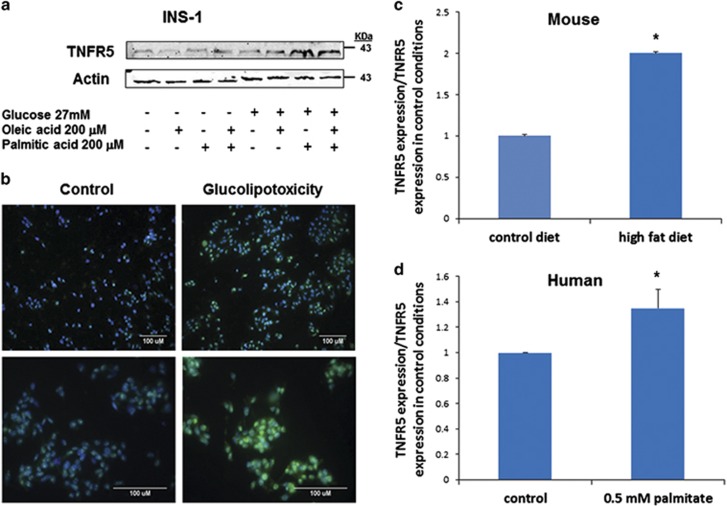
TNFR5 protein expression in INS-1 *β*-cells, high-fat-fed mouse islets, and human islets. (**a**) INS-1 cells incubated for 72 h in RPMI-1640 media supplemented with or without 28 mM glucose, 200 *μ*M oleic acid, and 200 *μ*M palmitic acid. Immunoblots were conducted using anti-TNFR5 primary antibody (Santa Cruz Biotechnology, Dallas, TX, USA) and Li-Cor IR secondary antibody (Lincoln, NE, USA), and are representative of three independent experiments. (**b**) Imuunofluorescence of INS-1 cells fixed in 4% paraformaldehyde for 30 min, then permeabilized with 0.1% Triton X-100. Samples were treated with anti-DAPI (blue; Life Technologies Ltd., Paisley, UK) or anti-TNFR5 primary antibody (green), and incubated with secondary antibody conjugated to fluorescence dye (AF488; Life Technologies Ltd.). Images were taken with a Leica epi-fluo microscope (Wetzlar, Germany) using either a 20 × objective (upper panels) or 40 × objective (lower panels), and in each case are representative of three independent experiments. (**c**) C57Bl/6 mice were fed a high-fat or standard rodent diet for 10 weeks. Islets of mice were extracted and digested with collagenase, RNA extracted, reverse transcribed, and qRT-PCR analysis performed. Data were analysed using the ΔΔ_CT_ method from four independent experiments. (**d**) Human islets were cultured 24 h with or without 0.5 mM palmitate. RNA was extracted, reverse transcribed, and qRT-PCR analysis performed. Data were analysed using the ΔΔ_CT_ method from six independent experiments. *P<0.05

Although our data indicate significant upregulation in *TNFRSF5* gene expression in *β*-cells exposed to a glucolipotoxic environment, mRNA does not always equate to protein. Therefore, we incubated INS-1 cells for 72 h in RPMI-1640 supplemented with either 28 mM glucose, 200 *μ*M oleic acid, or 200 *μ*M palmitic acid, both individually and in combination, and immunoblotted with anti-TNFR5 polyclonal antibody. The combination of glucose and fatty acids induced the strongest (two- to threefold; *P*<0.01) upregulation in TNFR5 protein (Figure 2a), although fatty acids or glucose alone also induced upregulation albeit by a more modest increase. These data are supported by immunofluorescence experiments (Figure 2b), in which there was a strong increase in TNFR5 intensity (green) in INS-1 cells incubated for 72 h in the presence of high glucose and fatty acids. Thus, a parallel increase in protein levels assessed both by western blot an immunofluorescence was detected under glucolipotoxic conditions.

INS-1 tissue culture cells offer a consistent response to experimental manipulation, which makes them an ideal choice for microarray analysis where large numbers of repeat experiments are costly. However, as transformed *β*-cell lines are not always fully representative of primary *β*-cell biology, it becomes important to determine whether these findings are indicative of whole animal physiology. Therefore, C57Bl/6 mice were fed a high-fat diet for 10 weeks, resulting in impaired glucose tolerance, diminished insulin secretion, and reduced islet size. Pancreas was removed and total RNA extracted from isolated islets. In line with our INS-1 cell data, qRT-PCR analysis of extracted RNA showed a twofold increase in *TNFRSF5* expression in the high-fat-fed mice relative to lean controls (Figure 2c). To determine whether our data were also reflective of human physiology, we next isolated islets post-mortem from human donors. These islets were incubated for 24 h in RPMI-1640 supplemented +/− 0.5 mM palmitate, total RNA extracted, and qRT-PCR performed. Despite the shorter incubation period, we again observed statistically significant upregulation of *TNFRSF5* (1.4-fold; *P*<0.05; Figure 2d), indicative of a similar pattern of upregulation of *TNFRSF5* in rodent and human islets.

**Figure 3:**
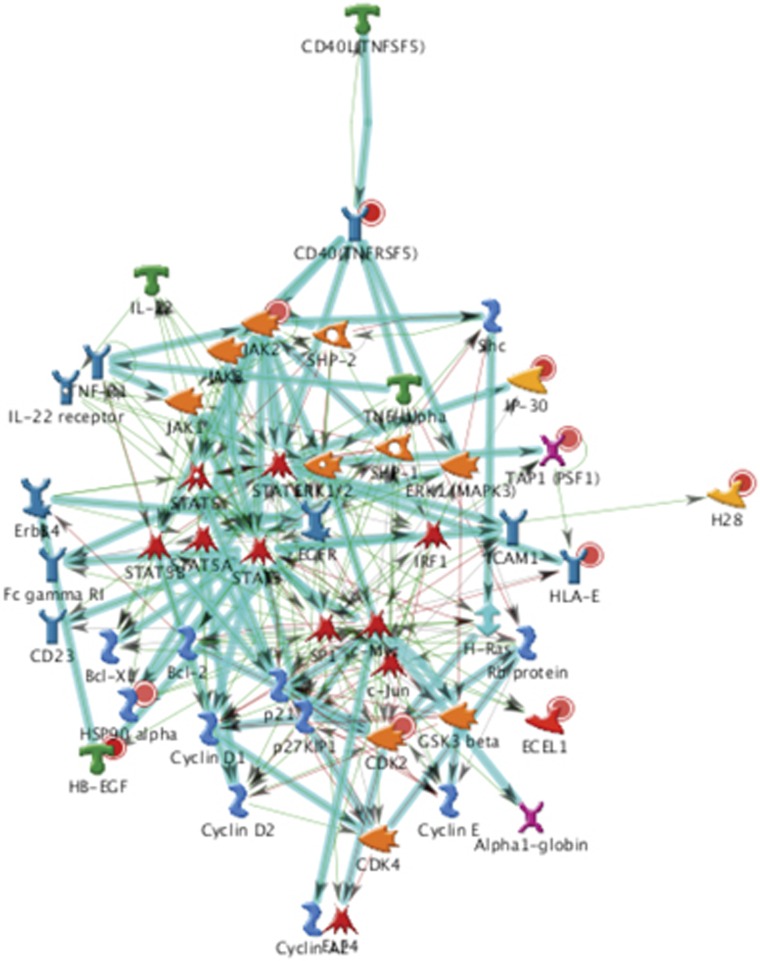
Network analysis of TNFR5 signalling. MetaCore (http://genego.com) was used to build a network from significantly upregulated genes in INS-1 cells subjected to glucolipotoxicity. The most significant biological network (*P*<0.001) included positive regulation of cellular, biological, and metabolic processes linked to TNFR5 and JAK/STAT signalling. Details of network objects and interactions are provided in the Metacore legend (Supplementary Appendix Figure 1). Thick cyan lines indicate the fragments of canonical pathways. Upregulated genes are marked with red circles

To identify wider TNFR5 pathway interactions, non-biased network analysis was performed using the MetaCore integrated knowledge database of pathways (https://portal.genego.com). The gene content from microarray analysis files was used as the input list for generation of biological networks, using the analyse network algorithm with default settings. This is a variant of the shortest paths algorithm with main parameters of (1) relative enrichment with the uploaded data and (2) relative saturation of networks with canonical pathways. CD40L signalling through TNFR5 came out as the top ranked of all upregulated networks irrespective of cellular function. A further major finding from this analysis was the central involvement of Janus kinase (JAK)/STAT signalling pathways (Figure 3). As JAK/STAT signalling is often also linked to additional cytokine signalling through interferon (IFN)-*γ*-related pathways, we were able to assess the likely validity of these predictions by going back to our original data set and determining whether IFN-related genes showed changed expression. Of the six genes that came through our bioinformatic filter with statistically significant data, all six were upregulated. Furthermore, upregulation of all six genes was further confirmed by independent PCR analysis and shown to be statistically significant (*P*<0.001; Supplementary Appendix Table 2).

**Figure 4:**
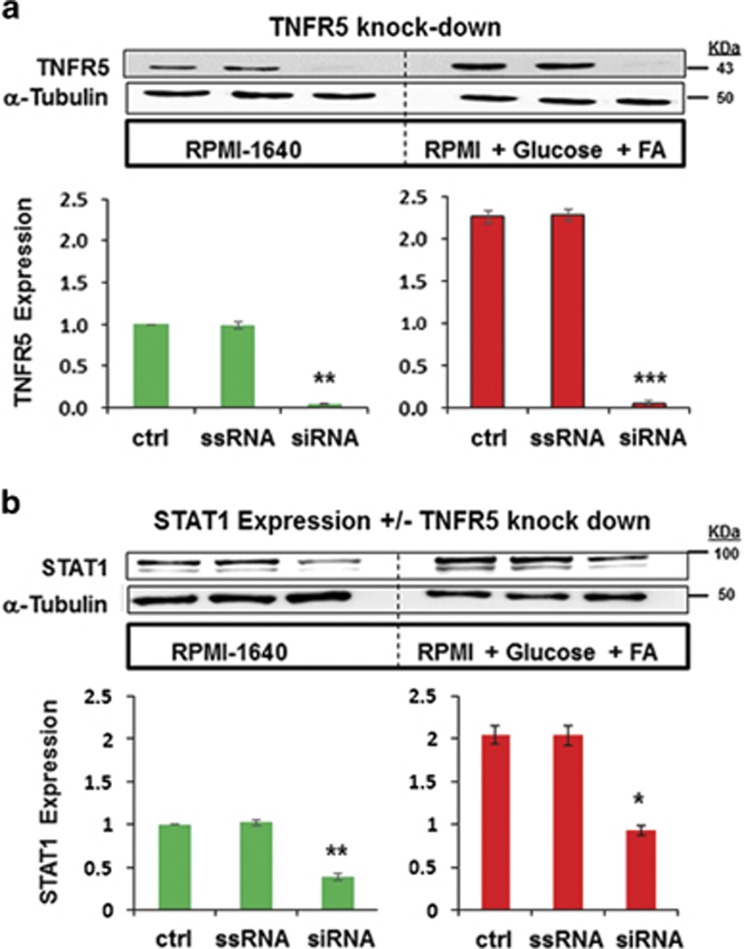
Effect of TNFR5 on STAT1 expression. (**a**) INS-1 cells were either mock-transfected, transfected with scramble sequence RNA (ssRNA), or with specific interfering oligonucleotide (siRNA). Cells were then incubated for 72 h in RPMI-1640 media supplemented with or without 28 mM glucose, plus 200 *μ*M oleic acid, and 200 *μ*M palmitic acid. Immunoblots were conducted using anti-TNFR5 primary antibody (Santa Cruz Biotechnology). Alpha-tubulin was used for normalisation of the data. TNFR5 protein expression fold-changes are expressed as mean±S.E.M. of data obtained from three different experiments. (**b**) INS-1 cells were either mock-transfected, transfected with ssRNA, or with siRNA oligonucleotide. Cells were then incubated for 72 h in RPMI-1640 media supplemented with or without 28 mM glucose, plus 200 *μ*M oleic acid, and 200 *μ*M palmitic acid. Immunoblots were conducted using anti-STAT1 primary antibody (Cell Signalling Technology, Beverly, MA, USA). Alpha-tubulin was used for normalisation of the data. Protein expression fold-changes are expressed as mean±S.E.M. from data obtained from three different experiments. *P<0.05, **P< 0.01, ***P< 0.001

To gain more detailed understanding of the contribution of TNFR5 in triggering glucolipotoxic STAT1 and NF-kB responses, we selectively modulated TNFR5 expression and activity, and determined the resulting effect on STAT1 and NF-*κ*B. INS-1 cells were transfected with either scramble sequence or short interfering RNA (siRNA) specifically directed against TNFR5, then incubated for a further 72 h in media supplemented with or without 28 mM glucose, 200 *μ*M oleic acid, and 200 *μ*M palmitic acid. Although the scramble sequence had no effect upon TNFR5 expression, the selective oligonucleotide brought down expression of TNFR5 by 96.0 +/− 1.0% in cells cultured in standard RPMI-1640, and by 97.8 +/− 1.3% in cells cultured in RPMI-1640 supplemented high glucose and fatty acids (Figure 4a). We then went on to determine STAT1 expression in these cells. Culturing cells for 72 h in media supplemented with high glucose and fatty acids resulted in STAT1 expression more than doubling (Figure 4b). However in cells where TNFR5 had been knocked down (siRNA lanes), STAT1 expression decreased by 61.0 +/− 4.0% in cells cultured in standard RPMI-1640 media (left panel), and by 54.6 +/− 2.9% in cells cultured in RPMI-1640 supplemented with high glucose and fatty acids (right panel).

**Figure 5:**
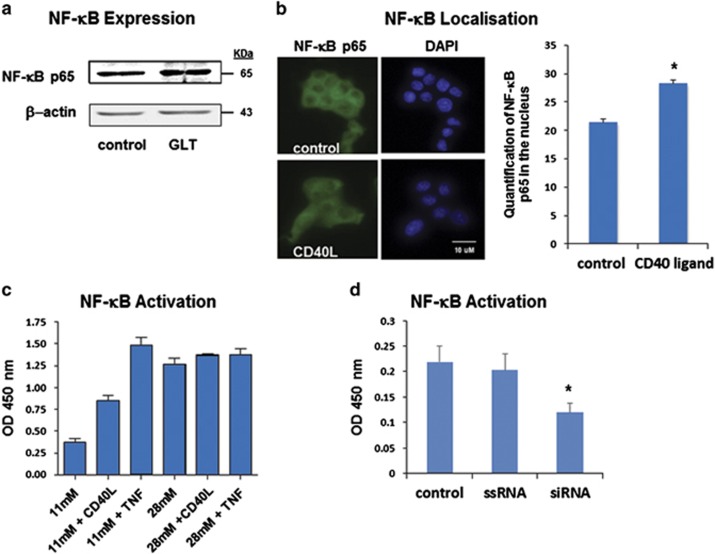
Effect of TNFR5 signalling on NF-*κ*B expression, localization, and activity. (**a**) INS-1 cells were incubated for 72 h in RPMI-1640 media supplemented with or without 28 mM glucose, plus 200 *μ*M oleic acid, and 200 *μ*M palmitic acid (GLT). Immunoblots were conducted using using anti-NF-*κ*B p65 subunit primary antibody (Santa Cruz Biotechnology). Beta-actin was used for normalization of the data. Data presented are representative of three independent experiments. (**b**) INS-1 cells were cultured onto coverslips and incubated with CD40 ligand (1 *μ*g/ml) for 6 h. Cells were then fixed and stained with anti-NF-*κ*B p65 polycolonal antibody (green) and DAPI (blue). Images were taken with a Leica EpiFluo microscope with a 63 × objective (representative image of three independent experiments). p65 translocation to the nucleus was quantified using Image J. (**c**) INS-1 rat pancreatic *β*-cells were cultured in RPMI-1640 media supplemented with glucose, TNF, or CD40 at the indicated concentrations and for the duration stated. NF-*κ*B subunit activity was detected using TransAm ELISA kit (Active Motif, Rixensart, Belgium) directed against NFκB p65 subunits. Absorbance was measured at 450 nm, with a reference wavelength of 655 nm. All experimental data were gathered from a series of three independent experiments. (**d**) INS-1 cells were either mock-transfected, transfected with ssRNA, or with siRNA oligonucleotide, and cells then incubated for 72 h in RPMI-1640 media. NF-kB activity was quantified as outlined above. Experimental data were gathered from a series of five independent experiments. *P<0.05

We next investigated how glucolipotoxicity and TNFR5 activity affect NF-*κ*B expression, localisation, and activity. Following 72 h incubation in media supplemented with 28 mM glucose and 200 *μ*M fatty acids, we observed a 73.5 +/− 5.7% increase in NF-*κ*B expression (Figure 5a). Similarly, 6 h exposure of INS-1 cells to 1 *μ*g/ml CD40L (the physiological activator of TNFR5) resulted in increased translocation of the NF-*κ*B p65 subunit to the nucleus (Figure 5b). Although this is consistent with NF-*κ*B activation by TNFR5, we nonetheless sought to confirm this using a quantitative ELISA assay that we have previously used to determine NF-*κ*B activity in nuclear extracts isolated from cells of patients with autoinflammatory fevers.^19, 20, 21^ Using this assay, we were also able to compare NF-*κ*B activity in *β*-cells treated with high glucose, TNF*α*, or CD40L. As can be seen (Figure 5c), 72 h incubation of INS-1 cells in 28 mM glucose resulted in an increased level of NF-*κ*B activity similar to that observed, following 2 h exposure of cells to 100 ng/ml of TNF*α*. However, under low-glucose conditions where TNFR5 expression is not upregulated beyond basal, 6 h exposure to 1 *μ*g/ml CD40L nevertheless still succeeded in eliciting half-maximal induction of NF-*κ*B activity.

The above experiments detail how glucolipotoxicity influences TNFR5 expression and activity. They do not however tell us whether the increased NF-*κ*B activation is dependent upon TNFR5, or is instead simply coincidental to increased TNFR5 expression and activity. To address this point, we again knocked down TNFR5 expression, then measured NF-*κ*B activity in mock-transfected cells, cells transfected with scramble sequence, and cells transfected with selective oligonucleotide. Although we were only able to achieve a much more modest knockdown of TNFR5 in this particular series of experiments (58.3 +/− 16.9%), this still significantly ablated NF-*κ*B activity by 46.7 +/− 8.2%.

## Discussion

Using an unbiased high-density microarray screen, we have been able to determine the effect of high glucose and fatty acid environment on thousands of genes simultaneously. Among the top 25 hits, our, data indicates the presence of disease association with both endocrine and metabolic disorders, which suggests the presence of pathways with known T2D and obesity aetiology in man. Interestingly, the molecules differentially expressed in this study were also found to be important in other diseases. Of those diseases were a number of brain disorders. This may reflect the pancreas being a highly innervated organ that shares a number of molecular similarities with brain at the level of transcriptome and proteome, the roots to which likely lie in the fact that the pancreas contains ancestral precursors of both pancreatic and neural crest origin.^22^ The data also indicate changed expression of genes associated with pathways common to many forms of neoplasm. Epidemiologic evidence suggests that people with diabetes are at significantly higher risk for many forms of cancer.^23^ Furthermore several studies indicate an association between diabetes and risk of liver, pancreatic, endometrial, colon/rectum, breast, and bladder cancer. Although common risk factors such as age, obesity, physical inactivity, and smoking undoubtedly contribute to the increased cancer risk in diabetic patients, hyperinsulinemia may also increase the likelihood of developing cancer due to insulin being a growth factor with metabolic and mitogenic effects. Its action in malignant cells is also favoured by mechanisms acting at both the receptor and post-receptor level.^24^

In agreement with the data presented here, it has previously been shown that both human and murine pancreatic *β*-cells express functional TNFR5.^25, 26^ Our observation that exposure of *β*-cells to high levels of glucose and/or fatty acids results in a two- to fourfold increase in expression of TNFR5 may therefore have clinical implications. However, without ligand activation increased receptor expression is unlikely to have a significant deleterious impact on functional *β*-cell mass. The pro-apoptotic response to glucose and lipids is not restricted to islet cells alone though. Previous work has shown that Indian subjects with diabetes have approximately three times higher soluble cluster of differentiation 40 ligand (sCD40L) levels in their plasma than individuals with normal glucose tolerance.^27^ The same study also found that plasma sCD40L levels were also elevated in people with impaired glucose tolerance, metabolic syndrome, and insulin resistance. Other researchers reported similar findings in a subsequent study in Europeans with T2D.^28^ Interestingly, this latter publication also reported a significant decrease in platelet TNFR5 signalling following sustained reduction in haemoglobin A1C, indicating that the process may be reversible. A positive correlation between glycaemic control and sCD40L level has also recently been reported in patients with type 1 diabetes.^29^

Our predictive pathway analysis indicates the potential for TNFR5 to regulate a wide range of cellular functions, including those that direct the ultimate fate of the *β*-cell – namely life and regeneration through cell proliferation (STAT5 and MAP3K) and cell cycle (cyclins and associated kinases), or cell death (Bcl-2 and B-cell lymphoma-extra large). At the heart of each of these pathways, however, lies NF-*κ*B and/or STAT1. Therefore, the combination of high sCD40L levels found in diabetes and the upregulated expression of functional *β*-cell receptor shown here suggests that TNFR5 signalling may be responsible for much of the glucolipotoxicty-induced NF-*κ*B activity in humans. Furthermore, given that NF-*κ*B^5, 6^ and STAT1^7^ activity are known to subsequently lead to islet cell death, we hypothesise that TNFR5 signalling likely represents a major cause of islet cell death in people with poorly controlled glucose homoeostasis and chronically elevated levels of glucose and fatty acids.

As glucolipotoxic induction of both STAT1 and NF-*κ*B expression and activity can be prevented by downregulation of TNFR5 expression this has major potential therapeutic implications, not least as it might be possible to enhance islet cell survival through targeted disruption of TNFR5 pathways. Anti-TNFR5 strategies have previously been employed as a general immunosuppressant strategy following transplantation, and also to treat people suffering from lupus and several types of cancer. Intervention has not always met with success however, as there are a number of reports where anti-CD40L monoclonal antibodies have been shown to induce thromboembolic events.^30, 31, 32, 33^ Importantly, these dangerous side effects have not been observed when the receptor, rather than ligand, is targeted. Indeed, there are now numerous highly promising clinical trials taking place, involving different anti-TNFR5 monoclonal antibodies.^34, 35^ Of these, those employing antagonistic anti-TNFR5 antibodies are of particular relevance to our current study. Lucatumumab is one such example that has been found to be well tolerated,^36^ at least in moderate and intermediate strength doses.^37^ Importantly, it has also been shown to prevent induction of NF-*κ*B activity in multiple myeloma cells.^38^ This strategy would need to be modified for the treatment of diabetes though, as immune cell interactions with the Fc region of cell-bound lucatumumab could potentially lead to either opsonisation of *β*-cells or antibody-dependent cell cytotoxicity. Phage display technology may provide an alternative solution however, facilitating the generation of antibody fragments containing the key heavy and light chain antigen recognition sites, although lacking the Fc region of the full molecule. This is an area that clearly warrants future research.

## Materials and Methods

### Materials

Antibodies were obtained from Santa Cruz Biotechnology Inc., Cell Signalling Technology, and Li-Cor Bioscience. Unless otherwise stated, all other chemicals were purchased from Sigma Aldrich (St. Louis, MO, USA) or VWR International Ltd (Lutterworth, UK).

### Cell culture and islet isolation

INS-1 *β*-cells was cultured in RPMI-1640 media supplemented where indicated with 28 mM glucose, 200 *μ*M oleic acid, or 200 *μ*M palmitic acid for 72 h as detailed previously.^39^ C57Bl/6 mice (Charles River, UK) were fed a high-fat (60% fat-58Y1; Test Diets, St. Lois, MO, USA) or standard rodent diet for 10 weeks. Pancreas was surgically removed and digested in Hank's buffered salt solution containing collagenase P (1 mg/ml) and DNAse I (0.15 mg/ml; both Roche Diagnostics, Burgess Hill, UK). All animal experiments were conducted in accordance with the UK Home Office Animals (Scientific Procedures) Act, 1986, with local ethical committee approval. Human islets were isolated from non-diabetic multiorgan donors as previously detailed,^40^ with approval of the local ethics committee and with written informed consent from family members. In both cases, islets were hand-picked into RPMI-1640 and immediately lysed for RNA or protein experiments.

### Quantitative RT-PCR

Cells were trypsinised, washed with cold phosphate-buffered saline (PBS) and lysed in RNAeasy QTL lysis buffer. Total RNA was prepared from lysed cells using RNeasy kit (Qiagen, Hilden, Germany) according to manufacturers recommended procedures (Qiagen). cDNA was generated from RNA using a standard RT kit (Promega, Madison, WI, USA). qRT-PCR reactions were performed using Maxima SYBR Green/ROX qPCR Master Mix (ThermoFisher Scientific, Loughborough, UK) and the following primers: rat CD40 forward primer GTCGGATTCTTCTCCAATG; rat CD40 reverse primer ACAGAGGGTATCAGTCTGAC; mouse CD40 forward primer TGGTCATTCCTGTCGTGATG; and mouse CD40 reverse primer GGCTCTGTCTTGGCTCATCT. Human islet experiments were performed as previously described,^41^ with CD40 oligonucleotide obtained from assay-on-demand gene expression products (Applied Biosystems, Foster City, CA, USA). Target gene mRNA was quantified and normalised for *β*-actin using an ABI7700 bioanalyzer (Applied Biosystems). Data were analysed using the ΔΔ_CT_ method.

### Affymetrix arrays

Quality and integrity of extracted RNA was assessed using an Agilent Bioanalyser 2100 (Agilent Technologies, Santa Clara, CA, USA). A measure of 10 *μ*g total RNA was converted into double-stranded cDNA using Superscript Reverse Transcriptase kit (Life Technologies Ltd.), following manufacturer's T7-(dT)-based recommendation. T7 RNA polymerase was then used to convert the cDNA to biotin-labelled cRNA. cRNA generated with T7 polymerase was chemically fragmented, then hybridized overnight at 45 °C to Affymetrix high-density GeneChip Rat Genome 230 2.0 arrays (Affymetrix), each containing 31 000 probe set, analysing over 30 000 transcripts and variants from over 28 000 well-substantiated rat genes following Affymetrix Expression Analysis Technical Manual procedures. Chips were then washed and stained with streptavidin-phycoerythrin (SAPE). Signals were amplified by incubation with biotinylated anti-streptavidin antibody (Vector Laboratories, Burlingame, CA, USA) followed by a final SAPE staining step. Fluorescent pixel intensities for each probe were determined using a confocal laser scanner (GeneArray Scanner, Affymetrix), processed, quantified, background adjusted, and scaled using Affymetrix Microarray Suite 5.0 software (Affymetrix). Further MAS 5.0 analysis (Affymetrix) of scaled intensity data generated the absolute analysis for each probe, as well as comparative expression reading between two GeneChip arrays (Affymetrix). Condition-to-condition differential expression profile analysis was carried out using Genomics Suite (Partek Incorporated, St.Louis, MO, USA). Principal component analysis was applied to identify any independent sources of variation in the data.

### Pathway identification and visualisation

For comprehensive analysis of biological pathways in which transcripts were differentially expressed we combined expression data from the microarray analysis with information in Reactome version 29, (http://www.reactome.org), a knowledgebase of biological pathways.^42^ To visualise the networks, we used MetaCore (http://genego.com) integrated knowledge database and software suite. Data analysis tools were used to generate high-resolution images of networks, with details of network objects and interactions provided in the Metacore legend (Supplementary Appendix Figure 1).

### Western blotting

INS-1 cells were lysed and protein separated by SDS-PAGE. Protein was then transferred to nitrocellulose as described previously.^39^ Protein was detected using either anti-STAT1 (Cell Signalling Technology), anti-TNFR5, or anti-NF-*κ*B p65 primary antibody (Santa Cruz Biotechnology), and Li-Cor IR secondary antibody. Antibody binding was detected using the LI-COR Fc Dual-Mode Imaging System (LI-COR).

### Immunofluoresence

Cells were seeded in 12-well plates onto coverslips for 72 h in different media conditions, then fixed in 4% paraformaldehyde in PBS for 30 min and subsequently washed three times in PBS and store at +4 °C overnight. The following day, cells were permeabilized by with 0.1% Triton X-100 in PBS for 5 min, washed in 0.1% Triton in PBS, then incubated in primary antibody for 2 h at room temperature. Coverslips were then washed three times in PBS+Triton X-100 and then incubated with secondary antibody conjugated to fluorescence dye (AF488, ThermoFisher Scientific) for 1 h at room temperature. Images have been taken using a Leica DM5000 epi-fluo microscope.

### Gene silencing

INS-1 cells were cultured to 40–50% confluency. siRNA oligonucleotide was combined with diluted lipofectamine RNAi max and mixed gently. After 25 min at room temp, the solution was mixed with RPMI and serum, but without antibiotics. This was then added to cells for 24 h, after which it was replaced with complete RPMI. Knockdown was detected 48–72 h after transfection by western blot.

### NF-*κ*B activity

TransAM NF-*κ*B Family Kits (Active Motif, Rixensart, Belgium) were utilised for the study of specific NF-*κ*B subunit pathways. In brief, oligonucleotide containing an NF-*κ*B consensus-binding site was immobilized onto 96-well plates. The binding of NF-*κ*B to its consensus sequence was detected by adding 30 *μ*l of complete binding buffer to each well and 1 *μ*g of nuclear extract diluted in complete lysis buffer was then added per well. The plates were sealed and incubated for 1 h at room temp with mild agitation. Each well was then washed three times with 200 *μ*l 1 × wash buffer. Antibody directed against one of NF-*κ*B p65 subunit was bound to the protein-oligonucleotide complex and detected following addition of secondary antibody, conjugated to horse radish peroxidase. Developing solution was added and the plate was incubated for 2–10 min at room temperature. Absorbance was then measured, using a spectrophotometer (Perkin Elmer-Wallac, Milan, Italy) Victor-1420 multi-label counter at 450 nm with a reference wavelength of 655 nm.

### Statistical analysis

Results are expressed as mean±S.E.M. (*n*=3 or more independent experiments). Parameters were compared using unpaired Student's *t*-test and a *P*-value of<0.05 was considered significant.

## Figures and Tables

**Table 1:** Identification of associated diseases

**Associated diseases**	**Ratio**	***P*-value**
Mental disorders	206/1593	1.212E−13
Psychiatry and psychology	233/1875	1.810E−13
Mood disorders	121/789	3.601E−13
Glioma	492/4765	6.519E−13
Neoplasms, neuroepithelial	511/4996	9.286E–13
Breast neoplasms	813/8688	3.199E–12
Breast diseases	813/8689	3.291E–12
Neoplasms, glandular and epithelial	808/8659	8.177E–12
Endocrine system diseases	564/5714	2.006E–11
Digestive system neoplasms	1044/11 780	1.513E–10
Skin diseases	993/11 125	1.770E–10
Rectal diseases	823/8967	2.047E–10
Gonadal disorders	334/3128	2.920E–10
Genital diseases, female	1129/12 925	3.174E–10
Pathological process	290/2642	3.419E–10
Astrocytoma	296/2709	3.514E–10
Genital neoplasms, female	1119/12 809	4.717E–10
Gastrointestinal neoplasms	940/10 509	8.836E–10
Ovarian neoplasms	313/2927	1.068E–09
Nutritional and metabolic diseases	328/3099	1.214E–09
Ovarian diseases	323/3046	1.370E–09
Adnexal diseases	323/3049	1.528E–09
Glioblastoma	286/2636	1.565E–09
Digestive system diseases	1071/12 231	1.672E–09
Intestinal neoplasms	839/9256	1.826E–09

INS-1 *β*-cells were incubated for 72 h in RPMI-1640 media supplemented with or without 28 mM glucose, plus 200 *μ*M oleic acid, and 200 *μ*M palmitic acid. RNA was extracted and hybridized to Affymetrix high-density microarrays (Affymetrix). Differentially expressed genes were determined using Partek software (Partek Inc., Chesterfield, MO, USA) based on a *P*<0.05 from three independent experiments. Predicted associated diseases were identified using MetaCore (http://genego.com) integrated knowledge database

**Table 2:** TNFR family member expression

	**TNFR1**	**TNFR5**	**TNFR6**
Affymetrix	—	2.23 (*P*<0.0001)	1.59 (*P*<0.05)
PCR	2.23 (*P*=NS)	3.62 (*P*<0.05)	1.19 (*P*=NS)

Abbreviations: NS, not significant; TNFR, tumour necrosis factor receptor

TNFR1, TNFR5, and TNFR6 expression was determined using Affymetrix array data and independent qRT-PCR analysis. Data are shown from three independent experiments

## Abbreviations

ER: endoplasmic reticulum
IFN: interferon
IL: interleukin
JAK: Janus kinase
NF-*κ*B: nuclear factor kappa-light-chain-enhancer of activated B cells
PBS: phosphate-buffered saline
qRT-PCR: quantitative real-time PCR
ROS: reactive oxygen species
RPMI: Roswell Park Memorial Institute
sCD40L: soluble cluster of differentiation 40 ligand
siRNA: short interfering RNA
STAT: signal transducer and activator of transcription
T2D: type 2 diabetes
TNFR: tumour necrosis factor receptor
