# Supplementary material for: Glucolipotoxicity initiates pancreatic β-cell death through TNFR5/CD40-mediated STAT1 and NF-κB activation
Source: Cell Death Dis. 2016 Aug 11;7(8):e2329–. doi: 10.1038/cddis.2016.203 (PMC5108311; doi:10.1038/cddis.2016.203)
Supplement: Supplementary Appendix Figure 1 [file cddis2016203x1.pdf]

Affy ID	Gene Symbol	Corr p-value	LOG Fold-Change
1390949_at	---	8.1700E-06	1.44
1386577_at	Klhl23	1.2000E-05	1.57
1377718_at	Fancb	1.4800E-05	1.67
1391277_at	S100a5	1.7500E-05	4.70
1368983_at	Hbegf	2.1200E-05	2.64
1388378_at	Eif3c	2.3800E-05	1.26
1377698_at	Cd40	3.1100E-05	2.23
1398590_at	---	4.4400E-05	1.33
1375855_at	Epdr1	4.4700E-05	1.20
1379757_at	---	6.7000E-05	1.49
1368686_at	Ambp	7.0900E-05	1.53
1382984_at	Tor1b	8.2100E-05	1.46
1397873_at	---	1.2096E-04	1.11
1372240_at	Sgca	1.2186E-04	1.30
1392516_a_at	Stard4	1.3068E-04	1.38
1389003_at	Rhobtb3	2.0143E-04	1.34
1376265_at	Steap2	2.1140E-04	1.30
1390118_at	Clp1	2.6854E-04	1.15
1379528_at	---	2.8421E-04	1.12
1371798_at	---	2.8444E-04	1.38
1389683_at	---	3.0736E-04	1.22
1388255_x_at	RT1-CE5	3.3196E-04	1.72
1371209_at	RT1-CE5	3.5861E-04	2.34
1387036_at	Hes1	3.6556E-04	1.69
1398481_at	---	3.7394E-04	1.20
1371998_at	Mobkl1b	3.8835E-04	1.12
1396373_at	---	3.9223E-04	1.20
1371142_at	Cyp2g1	3.9559E-04	1.20
1374157_at	Pde4b	3.9665E-04	1.69
1369633_at	Cxcl12	4.3709E-04	2.00
1377917_at	---	4.3993E-04	1.35
1383697_at	---	4.4309E-04	1.23
1387395_at	Adora2b	4.7163E-04	1.42
1374537_at	Chsy1	4.7997E-04	1.83
1371951_at	Fhl2	4.9229E-04	1.82
1376050_at	Taf5	5.2694E-04	1.41
1390864_at	---	5.2754E-04	1.33
1374304_at	Xrcc4	5.3550E-04	1.43
1389600_at	LOC363306 /// I	5.4484E-04	1.68
1393162_at	Slc39a6	5.6323E-04	1.93

1386946_at	Cpt1a	6.3023E-04	3.91
1391302_at	---	6.5084E-04	1.75
1388243_at	Gpr176	6.5584E-04	1.50
1376792_at	Fam176a	6.7988E-04	1.58
1373085_at	Cbr3	6.9075E-04	1.86
1393655_at	Rad54l	7.1992E-04	1.46
1386880_at	Acaa2	7.2252E-04	3.18
1367990_at	Crybb3	7.2478E-04	1.38
1385733_at	Exo1	7.5693E-04	1.57
1372133_at	Rras2	7.6477E-04	1.41
1389580_at	Hltf	7.6946E-04	1.43
1378496_at	---	7.7405E-04	2.29
1393598_at	Ccna1	8.0030E-04	1.22
1370928_at	Litaf	8.0211E-04	1.42
1374667_at	---	8.3537E-04	2.03
1376628_at	Zfp189	8.3808E-04	2.04
1367758_at	Afp	8.5195E-04	1.62
1370933_at	Myo1e	8.7282E-04	1.74
1378795_at	---	8.7462E-04	1.37
1383314_at	Tmem51	8.8550E-04	1.38
1379313_at	---	8.9927E-04	2.22
1389744_at	---	9.2790E-04	1.42
1383578_at	Rad51	9.3812E-04	1.61
1395898_at	---	9.3851E-04	1.23
1385086_at	Bub1	1.0079E-03	1.41
1391799_at	---	1.0223E-03	1.26
1376259_at	Prkcq	1.0854E-03	1.71
1370893_at	Acaca	1.1074E-03	1.86
1383398_at	RGD1564327	1.1147E-03	1.07
1398370_at	Adarb1	1.1359E-03	2.95
1376706_at	RGD1564799	1.1639E-03	1.39
1383148_at	---	1.1677E-03	1.37
1374872_at	Rasgrp2	1.2278E-03	2.82
1390026_at	Bag3	1.2605E-03	1.60
1377171_at	Lzts1	1.2831E-03	1.20
1375025_at	Camkk2	1.3144E-03	1.52
1375848_at	Pon2	1.3178E-03	1.33
1388552_at	Smpd1	1.3247E-03	1.39
1371796_at	Tmx2	1.3414E-03	1.25
1382685_at	Slit2	1.3576E-03	1.68
1379957_at	Slfn8	1.3807E-03	1.30

1390891_at	Kif11	1.3860E-03	1.56
1388856_at	Kitlg	1.4007E-03	4.25
1380365_at	---	1.4018E-03	1.22
1375552_at	Srp72	1.4261E-03	1.26
1380182_at	RGD1563437	1.4267E-03	1.26
1385808_at	Phf7	1.4287E-03	1.30
1376165_at	Slc24a3	1.4633E-03	2.39
1383233_at	Fam35a	1.4669E-03	1.23
1374649_at	Rasgrp2	1.4807E-03	6.79
1376249_at	Fuca2	1.4973E-03	1.47
1387834_at	Matk	1.5102E-03	2.61
1378296_at	Ncaph	1.5203E-03	1.33
1368260_at	Aurkb	1.5434E-03	1.45
1390383_at	Adfp	1.5593E-03	1.53
1383605_at	LOC360919 /// I	1.5918E-03	1.86
1396917_at	---	1.5987E-03	1.24
1372894_at	Tmem115	1.6065E-03	1.15
1389658_at	Nsun2	1.6495E-03	1.36
1397750_at	---	1.7492E-03	1.50
1388958_a_at	Slc2a4	1.7862E-03	3.52
1369859_at	Nr5a1	1.7965E-03	1.28
1374944_at	Znf691	1.8023E-03	1.06
1379676_a_at	Dnase1l1	1.8420E-03	1.24
1379914_at	Tcfcp2l2	1.8683E-03	2.11
1389226_at	Stag1	1.8763E-03	1.23
1382978_at	---	1.8958E-03	1.29
1378714_at	RGD1309873	1.9336E-03	3.22
1371943_at	Stk40	1.9430E-03	1.42
1393451_at	Cenpn	1.9460E-03	1.49
1376346_at	RGD1561797	1.9523E-03	1.52
1385170_at	Fstl5	2.0262E-03	4.24
1389396_at	---	2.0578E-03	1.58
1372450_at	---	2.0616E-03	1.32
1367818_at	Coq3	2.0732E-03	1.26
1399071_at	---	2.0943E-03	1.44
1394349_at	---	2.1116E-03	1.21
1382514_at	---	2.1511E-03	1.45
1377015_at	---	2.1910E-03	1.63
1372055_at	Prex1	2.1910E-03	1.55
1372372_at	Cmbl	2.1921E-03	2.06
1395494_at	---	2.2002E-03	1.14

1376624_at	---	2.2030E-03	1.77
1379420_at	RGD1565002	2.2198E-03	1.43
1392304_at	---	2.2235E-03	1.13
1382375_at	---	2.2334E-03	1.77
1391760_at	---	2.2480E-03	2.18
1391604_at	Ncapd3	2.2490E-03	1.32
1370962_at	Cipar1	2.2562E-03	2.56
1388706_at	RGD1308923	2.2571E-03	1.16
1368314_at	Ggcx	2.2662E-03	1.40
1367791_at	Ramp1	2.2938E-03	2.83
1378240_at	Fancd2	2.3014E-03	1.38
1374723_at	---	2.3358E-03	2.26
1376590_at	---	2.3398E-03	1.19
1383246_at	Armc7	2.3890E-03	1.35
1369559_a_at	Cd47	2.4420E-03	1.51
1378623_at	---	2.4692E-03	3.67
1373093_at	Errfi1	2.4865E-03	1.86
1393018_at	---	2.4897E-03	2.02
1378056_at	Gmnn	2.4937E-03	1.34
1372064_at	Cxcl16	2.4942E-03	2.25
1388807_at	Csrp2bp	2.5155E-03	1.32
1369520_a_at	Bcat1	2.5335E-03	1.18
1389711_at	---	2.5535E-03	1.29
1372870_at	Kdelr3	2.6003E-03	1.98
1368034_at	Chgb	2.6131E-03	1.35
1370973_at	Scn7a	2.6204E-03	2.42
1390415_at	Trip13	2.6659E-03	1.33
1368382_at	S100a3	2.6990E-03	3.60
1377955_at	---	2.7126E-03	1.50
1370438_at	Nos1ap	2.7178E-03	1.11
1395986_at	Slit2	2.7200E-03	1.57
1370977_at	Dpf1	2.7339E-03	1.36
1372595_at	Actn2	2.7346E-03	1.74
1393120_at	---	2.7493E-03	1.24
1373960_at	Tmem120a	2.8226E-03	1.28
1395982_at	Nup214	2.8725E-03	1.18
1388909_at	Oxnad1	2.8994E-03	1.17
1391027_at	---	2.9076E-03	1.25
1371631_at	Eif1ad	2.9102E-03	1.18
1380577_at	Abcg2	2.9403E-03	1.15
1388181_at	LOC641523	2.9403E-03	1.28

1396639_at	---	2.9725E-03	1.24
1369716_s_at	Lgals5 /// Lgals9	2.9763E-03	1.52
1397650_at	---	3.0019E-03	1.41
1384934_at	Slc41a2	3.0037E-03	1.35
1373803_a_at	Ghr	3.0326E-03	1.43
1391040_at	Rbbp8	3.0368E-03	1.51
1368706_at	Tm4sf4	3.0382E-03	1.53
1384567_at	---	3.0568E-03	1.14
1388395_at	G0s2	3.0793E-03	3.27
1385428_at	Xpo4	3.0834E-03	1.47
1390154_at	LOC686326	3.1017E-03	1.21
1384262_at	Ppp1r3b	3.1701E-03	2.32
1374493_at	---	3.1802E-03	1.73
1368882_at	St6galnac3	3.1851E-03	1.44
1387276_at	Dclk1	3.1958E-03	1.86
1383861_at	---	3.1960E-03	1.30
1373662_at	Tor2a	3.1970E-03	1.18
1397341_at	Pbk	3.2183E-03	1.38
1373909_at	LOC689176	3.2511E-03	1.33
1384280_at	Nusap1	3.2892E-03	1.57
1371970_at	Fam111a	3.3111E-03	1.35
1373049_at	RGD1562136	3.3310E-03	1.46
1382778_at	Dusp6	3.3500E-03	1.88
1375909_at	Gstm4	3.3547E-03	1.15
1376229_at	Foxo3	3.3612E-03	1.14
1387115_at	Ikbkap	3.3746E-03	1.16
1384948_at	---	3.3820E-03	1.30
1395020_at	Plekhh1	3.3828E-03	1.28
1392901_at	Lrrc1	3.3941E-03	1.34
1384087_at	---	3.3984E-03	1.22
1368432_a_at	Ros1	3.4001E-03	1.55
1373078_at	Ahcyl2	3.4722E-03	1.56
1378191_at	Ncbp1	3.4841E-03	1.45
AFFX_Rat_GAPD	Gapdh	3.4915E-03	1.09
1368526_at	Pex3	3.4965E-03	1.51
1370189_at	Sfrs10	3.5218E-03	1.21
1388598_at	LOC691995	3.5577E-03	2.19
1376893_at	Nrsn1	3.5656E-03	1.26
1373740_at	---	3.5715E-03	1.66
1368674_at	Pygl	3.5725E-03	1.81
1383047_at	Gas6	3.5746E-03	1.47

1373008_x_at	Rtn4r	3.6057E-03	1.60
1390384_at	H2afx	3.6122E-03	1.52
1372840_at	Zbed4	3.6986E-03	1.34
AFFX_ratb1/X12	---	3.7137E-03	1.18
1398969_at	---	3.7261E-03	1.26
1376317_at	Orc6l	3.7550E-03	1.35
1383886_at	---	3.7607E-03	2.12
1398000_at	---	3.8134E-03	1.23
1379092_at	---	3.8135E-03	1.90
1390801_at	RGD1359349	3.8271E-03	1.63
1369148_at	Hnf1a	3.8312E-03	1.72
1388407_at	RGD1311925	3.8333E-03	1.13
1378170_at	Aff4	3.8752E-03	1.30
1396492_at	Nfam1	3.8782E-03	1.40
1392683_at	---	3.8918E-03	1.11
1370108_a_at	Lin7a	3.8926E-03	2.38
1370963_at	Gas7	3.9060E-03	3.69
1398360_at	LOC679532	3.9265E-03	1.17
1383058_at	---	3.9637E-03	2.27
1371131_a_at	Txnip	3.9698E-03	2.31
1372828_at	Msrb2	4.0043E-03	1.56
1389756_at	Melk	4.0070E-03	1.37
1372341_at	Slc25a36	4.0244E-03	1.28
1373108_at	Ppp1r3c	4.0524E-03	3.82
1370940_at	Tjp2	4.0732E-03	1.47
1391205_at	Heatr2	4.0778E-03	1.93
1389052_at	Ttc13	4.1155E-03	1.20
1367698_a_at	Sep-09	4.1291E-03	1.37
1382028_at	Pex19	4.1336E-03	1.26
1376191_at	Hpgd	4.1367E-03	1.69
1378906_at	RGD1559690	4.1748E-03	1.73
1368229_at	Sip1	4.1986E-03	1.28
1391074_at	Crabp1	4.2017E-03	2.51
1389440_at	---	4.2185E-03	1.48
1374776_at	Vash2	4.2628E-03	1.95
1373870_at	Fam98a	4.3023E-03	1.13
1392461_at	Prpf4	4.3383E-03	1.20
1367846_at	S100a4	4.3543E-03	6.45
1393336_at	Swap70	4.3685E-03	1.27
1369687_at	Kcnab3	4.3710E-03	2.93
1372224_at	Chmp7	4.3730E-03	1.11

1393982_at	Pole2	4.3994E-03	1.39
1391181_at	---	4.4011E-03	1.34
1379969_at	Foxj2	4.4247E-03	1.28
1368470_at	Ggh	4.4399E-03	1.55
1392471_at	---	4.4416E-03	1.25
1373437_at	Ppp1r8	4.4459E-03	1.30
1373860_at	Sox4	4.4482E-03	1.99
1390407_at	Cldnd1	4.4807E-03	1.24
1380003_at	---	4.4918E-03	1.63
1372219_at	Tpm2	4.5282E-03	1.44
1380320_at	Lin54	4.6074E-03	1.32
1373235_at	Syap1	4.6320E-03	1.27
1372990_at	Creb3	4.6345E-03	1.25
1382225_at	---	4.6358E-03	2.72
1371392_at	Gpi	4.6535E-03	1.33
1368785_a_at	Pitx2	4.6680E-03	1.94
1398382_at	Nat13	4.7164E-03	1.43
1382592_at	Ccdc99	4.7174E-03	1.31
1376196_a_at	Med4	4.7737E-03	1.32
1379616_at	---	4.8075E-03	1.09
1368379_at	Scarb2	4.8297E-03	1.52
1391553_at	---	4.8455E-03	1.18
1388318_at	Pgk1	4.8517E-03	1.22
1395460_at	Caskin2	4.9614E-03	1.10
1376644_at	Med19	4.9785E-03	2.05
1384701_at	---	4.9954E-03	1.46
1397811_at	RGD1311723	5.0028E-03	1.28
1379748_at	H28	5.0501E-03	1.74
1373685_at	Ankrd37	5.0539E-03	1.22
1384364_at	Fbxo4	5.0546E-03	1.18
1374143_at	Epha2	5.0639E-03	1.33
1390321_at	RGD1304693	5.1493E-03	1.47
1392854_at	RGD1564560	5.1529E-03	1.47
1377975_at	---	5.1590E-03	1.50
1398249_at	Slc25a20	5.1892E-03	1.42
1385589_at	LOC679958	5.2172E-03	3.77
1380338_at	---	5.2180E-03	2.54
1372854_at	Ttc17	5.2233E-03	1.50
1388217_a_at	Calu	5.2471E-03	1.53
1367776_at	Cdc2	5.2484E-03	1.34
1383020_at	Cntrob	5.2676E-03	1.26

1395104_at	---	5.3060E-03	1.45
1389813_at	---	5.3064E-03	2.46
1383740_at	---	5.3249E-03	1.40
1392747_at	---	5.3410E-03	1.31
1368422_at	Meox2	5.3707E-03	1.03
1384861_at	---	5.3965E-03	2.61
1377666_at	Chdh	5.4123E-03	3.09
1373905_at	Hnrnpr	5.4324E-03	1.27
1382797_at	RGD1560433	5.4454E-03	1.50
1376829_at	---	5.4993E-03	3.27
1372027_at	---	5.5169E-03	1.74
1382388_at	Tmem143	5.5590E-03	1.15
1379813_at	RGD1565689	5.5864E-03	1.18
1390968_at	RGD1559643	5.6829E-03	1.08
1387721_at	Adora1	5.6948E-03	1.31
1373950_at	Znf496	5.7015E-03	1.26
1380277_at	Rad51ap1	5.7056E-03	1.42
1369686_at	Dclk1	5.7480E-03	1.72
1394842_at	Tmem19	5.7768E-03	1.38
1378801_at	---	5.7947E-03	1.57
1381572_at	RGD1565787	5.7977E-03	1.22
1372606_at	---	5.8592E-03	2.05
1382739_at	Gpr19	5.8736E-03	1.42
1379226_at	Aim1l	5.8736E-03	1.54
1390805_at	LOC689755	5.9121E-03	1.28
1394932_at	---	5.9149E-03	1.12
1367671_at	Pcna	5.9322E-03	1.16
1374035_at	Rem2	5.9790E-03	1.70
1380739_at	---	5.9920E-03	2.95
1367557_s_at	Gapdh /// Gapd	6.0368E-03	1.09
1370842_at	Bckdk	6.1210E-03	1.32
1381158_at	Spdya	6.1275E-03	1.21
1379448_at	Ttk	6.1571E-03	1.60
1391823_at	---	6.1697E-03	1.34
1378172_at	---	6.1992E-03	1.28
1369155_at	Cntn4	6.2252E-03	1.32
1387559_at	Grin3b	6.2487E-03	1.09
1397381_at	---	6.2543E-03	1.24
1389326_at	Rfc3	6.2607E-03	1.45
1376804_at	Myo6	6.2653E-03	1.46
1388510_at	Chmp6	6.2712E-03	1.20

1393478_at	---	6.2730E-03	1.31
1385477_at	RGD1563155	6.2850E-03	1.15
1373026_at	Spc24	6.3320E-03	1.54
1367701_at	Ramp2	6.3502E-03	1.34
1384479_at	Galnt3	6.3761E-03	1.38
1392652_at	---	6.4188E-03	1.18
1370180_at	Nudt4	6.4328E-03	1.28
1374694_at	Ankrd28	6.5168E-03	1.48
1389388_at	---	6.5192E-03	1.92
1383475_at	---	6.5440E-03	1.42
1391612_at	Il22ra2	6.5512E-03	1.17
1389103_at	Gins4	6.5642E-03	1.41
1389706_at	---	6.6003E-03	1.71
1380445_at	---	6.6355E-03	1.65
1376845_at	isg12(b)	6.6875E-03	2.39
1391586_at	---	6.6881E-03	1.11
1376000_at	---	6.6964E-03	2.51
1388212_a_at	H2-T23 /// H2-T	6.7020E-03	1.39
1370813_at	Gstm5	6.7153E-03	1.98
1393660_at	Acer1	6.7745E-03	1.72
1387195_at	St14	6.8170E-03	1.86
1386987_at	Il6ra	6.8223E-03	1.25
1381533_at	Rnd1	6.8438E-03	1.59
1371470_at	Adh4 /// Adh5	6.8600E-03	1.36
1385622_at	---	6.8730E-03	1.14
1389486_at	---	6.8997E-03	1.45
1373480_at	Hspa12a	6.9235E-03	2.84
1388404_at	Rpo1-3	6.9342E-03	1.26
1371115_at	Ptpre	6.9495E-03	1.65
1374869_at	---	7.0084E-03	1.26
1383370_at	Fam5b	7.1100E-03	1.86
1372524_at	---	7.1447E-03	1.21
1384244_at	Hsdl2	7.1472E-03	1.30
1375845_at	Aig1	7.1497E-03	1.42
1374478_at	RGD1305347	7.1952E-03	1.58
1376960_at	---	7.1976E-03	1.13
1387812_at	Pcsk6	7.1987E-03	2.13
1398182_at	---	7.2025E-03	1.26
1368469_at	Aqp5	7.2856E-03	2.44
1373568_at	---	7.2900E-03	1.33
1387891_at	Prdx4	7.3118E-03	1.25

1372776_at	Fbxl5	7.3460E-03	1.20
1392925_at	ST7	7.4341E-03	1.42
1387263_at	Pklr	7.4391E-03	1.93
1387957_a_at	Sh3kbp1	7.4867E-03	1.96
1373185_at	Ssr2	7.4932E-03	1.31
1391075_at	Rgs17	7.5660E-03	1.68
1390391_at	---	7.5963E-03	2.16
1385291_a_at	Sc65	7.6045E-03	1.31
1375247_at	Mgll	7.6563E-03	3.06
1377071_at	---	7.6752E-03	1.10
1369263_at	Wnt5a	7.6956E-03	1.33
1387244_at	Cgrrf1	7.7676E-03	1.23
1390244_at	Spats2	7.7851E-03	1.22
1388727_at	Cdc26	7.9057E-03	1.17
1391670_at	---	7.9280E-03	1.36
1390507_at	Isg20	7.9411E-03	1.41
1388827_at	H2afv /// LOC68	7.9803E-03	1.06
1379483_at	Bhlhe40	7.9987E-03	2.19
1388908_at	Peci	8.0399E-03	2.30
1389170_at	Casp7	8.0988E-03	1.50
1382811_at	---	8.1107E-03	1.19
1370419_a_at	Sh3kbp1	8.1183E-03	1.87
1391653_at	Gabrg2	8.1261E-03	1.40
1381897_at	Chst8	8.1328E-03	3.29
1381899_at	---	8.1421E-03	1.09
1398608_at	Cenpl	8.2009E-03	1.35
1374885_at	Slc12a6	8.2397E-03	1.50
1384980_at	---	8.2418E-03	1.93
1377457_a_at	Sorl1	8.2436E-03	1.41
1393165_at	Tmem206	8.2794E-03	1.22
1377168_at	Cpne9	8.2920E-03	4.32
1388977_at	Cept1	8.3319E-03	1.28
1389110_at	---	8.3571E-03	1.38
1384988_at	Fbxo5	8.3788E-03	1.32
1380243_at	RGD1304693	8.4074E-03	1.45
1374344_at	Prg-2	8.4530E-03	2.15
1376502_at	RGD1309228	8.5244E-03	1.35
1388825_at	---	8.5308E-03	1.51
1393824_at	RGD1559505	8.5634E-03	1.18
1379576_at	---	8.5760E-03	1.68
1389903_at	Pttg1ip	8.5832E-03	1.12

1383133_at	---	8.5942E-03	1.18
1389126_at	Chchd1	8.6023E-03	1.25
1376627_at	---	8.6132E-03	1.67
1367989_at	Slc2a4	8.6210E-03	3.81
1372343_at	Exosc8	8.6515E-03	1.41
1373348_at	LOC687424	8.6672E-03	1.63
1380504_at	Acaa2	8.6959E-03	2.19
1398847_at	Nudt4	8.7016E-03	1.14
1379214_at	---	8.7178E-03	1.60
1368991_at	Smpd3	8.7525E-03	1.58
1397745_at	---	8.7605E-03	1.11
1391137_at	RGD1359334	8.7822E-03	1.12
1372117_at	Mtmr10	8.9596E-03	1.46
1372761_at	---	8.9682E-03	1.20
1391142_at	---	8.9720E-03	1.30
1391678_at	Pax1	8.9738E-03	1.04
1368852_at	Dnaja1	8.9835E-03	1.14
1372242_at	Ddx3x	9.0155E-03	1.14
1374670_at	RGD1307722	9.0589E-03	1.65
1367897_at	Acadvl	9.0953E-03	1.33
1386178_at	---	9.1068E-03	1.19
1399109_at	---	9.1136E-03	1.28
1387349_at	Shox2	9.1622E-03	2.04
1373641_at	---	9.2031E-03	1.22
1384806_at	---	9.2433E-03	1.74
1384242_at	---	9.2458E-03	1.28
1399161_a_at	Erap1	9.3999E-03	1.39
1390358_at	Cacna2d3	9.4163E-03	1.37
1381475_at	Sdk2	9.4389E-03	1.19
1398778_at	Psma1	9.4396E-03	1.06
1387625_at	Igfbp6	9.4509E-03	1.34
1398108_at	Arhgdia	9.4928E-03	1.23
1377132_at	---	9.5040E-03	1.26
1398809_at	Nde1	9.5509E-03	1.38
1384090_at	Pigo	9.5556E-03	1.21
1389911_at	Metrnl	9.5653E-03	1.32
1388276_at	Hist1h3f	9.5724E-03	1.66
1383343_at	LOC690559	9.6010E-03	1.30
1383479_at	---	9.6064E-03	1.49
1389453_at	Rdm1	9.6413E-03	1.49
1368280_at	Ctsc	9.6685E-03	1.44

1379260_at	Heca	9.6763E-03	1.38
1367735_at	Acadl	9.7021E-03	1.67
1371845_at	Pop4	9.7258E-03	1.31
1376248_at	Sult2b1	9.8207E-03	1.82
1373250_at	Anln	9.8270E-03	1.27
1383980_at	---	9.8617E-03	2.27
1383433_at	Klhl23	9.8864E-03	1.41
1393541_at	---	9.8970E-03	1.22
1373808_at	---	9.9054E-03	1.37
1398273_at	Efna1	9.9264E-03	1.95
1390478_at	Orc4	1.0001E-02	1.40
1378387_at	Tinf2	1.0062E-02	1.33
1379694_at	Epc2	1.0063E-02	1.26
1371632_at	Coro1c	1.0149E-02	1.69
1389435_at	Fam114a1	1.0166E-02	1.88
1380619_at	RGD1305537	1.0183E-02	1.22
1395899_at	---	1.0194E-02	1.16
1374700_at	---	1.0195E-02	1.48
1393356_at	---	1.0247E-02	1.48
1383024_at	---	1.0264E-02	1.19
1368294_at	Dnase1l3	1.0274E-02	1.11
1370416_at	Mxd3	1.0293E-02	1.96
1392507_at	---	1.0305E-02	1.26
1377145_at	Gpr98	1.0310E-02	1.50
1372849_at	---	1.0362E-02	1.18
1379235_x_at	Cdc45l	1.0373E-02	1.42
1385781_at	Ercc6l	1.0378E-02	1.45
1393894_at	Cyp4a8	1.0393E-02	4.13
1369622_at	Prok2	1.0402E-02	4.23
1377884_at	---	1.0429E-02	1.43
1388915_at	---	1.0446E-02	1.12
1377599_at	Lpin1	1.0513E-02	1.16
1388017_at	Mamdc4	1.0576E-02	1.23
1370020_at	Slc25a10	1.0581E-02	1.56
1397882_at	Zbtb8a	1.0590E-02	1.37
1384220_at	Tbcc	1.0599E-02	1.12
1373633_at	---	1.0609E-02	1.41
1395626_at	---	1.0640E-02	1.26
1371996_at	---	1.0669E-02	1.30
1374329_at	---	1.0741E-02	1.28
1390707_at	Rgs10	1.0762E-02	1.98

1369149_at	Limk1	1.0845E-02	1.17
1378603_at	---	1.0854E-02	2.01
1384606_at	LOC100158225	1.0870E-02	1.29
1381196_at	Tm6sf2	1.0932E-02	2.91
1391588_at	---	1.0944E-02	1.51
1396948_at	---	1.0955E-02	1.19
1372181_at	Rpa1	1.0975E-02	1.25
1372417_at	Sertad1	1.0989E-02	1.46
1385007_at	Zcchc9	1.1002E-02	1.22
1396534_at	---	1.1018E-02	1.06
1368917_at	Nudt1	1.1023E-02	1.44
1384525_at	Dock11	1.1070E-02	1.20
1372062_at	Cinp	1.1072E-02	1.32
1374558_at	---	1.1081E-02	2.00
1389083_at	Smek2	1.1101E-02	1.32
1368650_at	Klf10	1.1103E-02	1.73
1368980_at	Plce1	1.1148E-02	1.73
1376722_at	Nup205	1.1152E-02	1.39
1388822_at	Scoc	1.1155E-02	1.11
1370268_at	Kcna5	1.1179E-02	1.54
1372071_at	Cd320	1.1229E-02	1.06
1378104_at	RGD1307465	1.1229E-02	1.25
1389735_at	Rps6ka6	1.1277E-02	1.55
1372102_at	Ncor1	1.1348E-02	1.28
1374194_at	---	1.1393E-02	1.31
1371924_at	Olfml3	1.1428E-02	1.86
1388103_at	Tmem37	1.1449E-02	1.22
1393092_at	Zfp53	1.1465E-02	1.22
1385068_at	---	1.1510E-02	1.52
1387086_at	Camlg	1.1535E-02	1.16
1368467_at	Cyp4f1	1.1556E-02	2.63
1395763_at	---	1.1580E-02	1.26
1389287_at	---	1.1592E-02	1.32
1368158_at	Scfd1	1.1618E-02	1.34
1380305_at	nod3l	1.1623E-02	2.60
1378320_at	Rlbp1	1.1658E-02	1.34
1382517_at	---	1.1746E-02	1.75
1381130_at	Mcm8	1.1747E-02	1.52
1390786_at	Arhgef2	1.1762E-02	1.86
1385196_at	---	1.1788E-02	1.30
1393647_at	Hat1	1.1797E-02	1.36

1373236_at	Ppcdc	1.1800E-02	1.32
1368079_at	Pdk1	1.1808E-02	1.87
1384501_at	---	1.1820E-02	1.72
1387732_at	Mterf	1.1828E-02	1.22
1389582_at	LOC683983	1.1830E-02	1.60
1380868_at	---	1.1845E-02	1.18
1388730_at	Cdc42ep4	1.1936E-02	1.16
1370157_at	Pln	1.1978E-02	1.21
1368080_at	Rgc32	1.1986E-02	2.26
1373027_at	Mllt4	1.2000E-02	1.15
1389572_at	Me3	1.2008E-02	1.91
1392287_at	---	1.2017E-02	1.18
1394654_at	Zfp451	1.2083E-02	1.22
1381084_at	---	1.2113E-02	1.20
1384762_at	---	1.2128E-02	1.45
1389668_at	Spc25	1.2152E-02	1.58
1376684_at	Dlgap5	1.2234E-02	1.38
1397784_at	---	1.2260E-02	1.43
1390763_at	---	1.2300E-02	3.22
1389617_at	Elk3	1.2329E-02	1.20
1382441_at	Arid1b	1.2336E-02	1.37
1393376_at	Sox6	1.2368E-02	1.23
1376304_at	---	1.2371E-02	1.71
1376226_at	---	1.2379E-02	1.81
1397630_at	Hecw2	1.2382E-02	1.76
1378072_at	Atad5	1.2423E-02	1.48
1383137_at	Sox4	1.2473E-02	2.44
1371363_at	Gpd1	1.2478E-02	2.12
1383529_at	---	1.2551E-02	1.37
1398602_at	Mad2l1	1.2572E-02	1.38
1384679_at	LOC679958	1.2575E-02	3.06
1377064_at	Dusp6	1.2662E-02	1.95
1396834_at	---	1.2703E-02	1.11
1393086_at	Uba6	1.2713E-02	1.38
1398202_at	---	1.2757E-02	1.27
1380943_at	RGD1311648	1.2769E-02	1.11
1397654_at	---	1.2820E-02	1.39
1394334_at	Chst8	1.2831E-02	3.62
1392739_a_at	Eepd1	1.2895E-02	2.24
1375210_at	RGD1307394	1.2927E-02	1.20
1371684_at	Pelo	1.2972E-02	1.25

1392573_at	Ube2a	1.3014E-02	1.34
1394435_at	Vangl1	1.3066E-02	1.68
1381036_at	---	1.3079E-02	1.40
1382743_at	LOC641315	1.3109E-02	1.32
1393081_at	Rasgef1a	1.3133E-02	1.78
1380068_at	---	1.3151E-02	1.10
1373506_at	LOC687750	1.3262E-02	1.51
1379451_at	---	1.3281E-02	1.90
1376132_at	Gtdc1	1.3320E-02	1.37
1379236_at	---	1.3449E-02	1.42
1388656_at	---	1.3507E-02	1.09
1372769_at	Lgtn	1.3507E-02	1.23
1380890_at	RGD1307055	1.3565E-02	1.07
1394403_at	Spata20	1.3604E-02	1.47
1372016_at	Gadd45b	1.3633E-02	1.50
1368037_at	Cbr1	1.3656E-02	1.24
1391610_at	---	1.3659E-02	1.12
1397797_at	Tigd3	1.3660E-02	1.38
1382830_at	Suv39h2	1.3727E-02	1.42
1392536_at	---	1.3730E-02	1.25
1376951_at	Mad2l1	1.3741E-02	1.36
1383366_at	---	1.3743E-02	1.29
1390818_at	---	1.3786E-02	1.22
1376711_at	Cldn11	1.3817E-02	1.20
1385526_at	Atg5	1.3825E-02	1.29
1388135_at	Rpa2	1.3831E-02	1.33
1380292_at	---	1.3834E-02	1.17
1385405_at	Adam23	1.3911E-02	1.26
1376625_at	LOC691862	1.3928E-02	1.16
1388970_at	Rasip1	1.3930E-02	1.23
1371352_at	Hmgn2	1.4009E-02	1.13
1378105_at	---	1.4017E-02	1.21
1380813_at	---	1.4030E-02	1.42
1392856_at	Serf1	1.4076E-02	1.55
1375266_at	---	1.4117E-02	1.70
1372650_at	Dnmbp	1.4274E-02	1.41
1379833_at	Lingo4 /// Rorc	1.4291E-02	1.83
1389666_at	Rom1	1.4296E-02	1.40
1387035_a_at	Arhgap17	1.4307E-02	1.14
1371718_at	Sra1	1.4357E-02	1.20
1374360_at	Cdc20	1.4372E-02	1.39

1389179_at	Cidea	1.4381E-02	1.24
1394490_at	Abca1	1.4388E-02	1.45
1370420_at	Srd5a1	1.4408E-02	1.49
1388591_at	Dclre1b	1.4436E-02	1.33
1376719_at	Tmem38b	1.4460E-02	1.28
1392659_at	Med31	1.4496E-02	1.64
1383010_at	---	1.4509E-02	1.19
1377137_at	Tdp1	1.4559E-02	1.43
1393933_at	Sorl1	1.4575E-02	1.31
1392034_at	---	1.4617E-02	1.18
1375984_at	Zfhx4	1.4621E-02	1.27
1370375_at	Gls2	1.4648E-02	1.17
1389988_at	Kctd2	1.4656E-02	1.45
1376489_at	Sos2	1.4714E-02	1.40
1391994_at	---	1.4717E-02	2.37
1390385_at	Glce	1.4719E-02	1.34
1371884_at	Ttc3	1.4723E-02	1.15
1398371_at	Ccdc132	1.4743E-02	1.15
1387527_at	Syngr1	1.4761E-02	1.38
1394280_at	Rgs17	1.4778E-02	1.47
1388149_at	Tap1	1.4789E-02	1.22
1378359_at	Nkrf	1.4817E-02	1.21
1391032_at	Sez6	1.4869E-02	1.72
1388963_at	Astn1	1.4871E-02	1.26
1376377_at	Diaph3	1.4997E-02	1.50
1390628_at	Cpeb2	1.5047E-02	1.63
1373179_at	LOC689994	1.5056E-02	1.38
1370348_at	Ninj1	1.5103E-02	1.64
1389705_at	Rag1ap1	1.5172E-02	1.84
1379900_at	---	1.5187E-02	1.36
1367772_at	Clns1a	1.5226E-02	1.22
1383642_at	---	1.5235E-02	1.31
1381933_at	Rfc5	1.5317E-02	1.41
1376589_at	LOC689540	1.5323E-02	1.26
1371582_at	Ilf2	1.5328E-02	1.48
1391919_at	Tcerg1l	1.5340E-02	1.38
1379218_at	Nlk	1.5368E-02	1.30
1369581_at	Pemt	1.5387E-02	1.20
1394988_at	---	1.5421E-02	1.18
1370855_at	Cst3	1.5436E-02	1.12
1374856_at	Bcdin3d	1.5444E-02	1.43

1370031_at	Gosr2	1.5459E-02	1.29
1394097_at	---	1.5492E-02	2.11
1371379_at	RGD1563422	1.5512E-02	1.34
1376193_at	---	1.5514E-02	1.39
1393768_at	Lingo1	1.5571E-02	1.24
1379377_at	Gdap2	1.5576E-02	1.30
1387136_at	Ptprv	1.5581E-02	1.45
1398483_at	Rgs17	1.5583E-02	1.77
1383879_at	---	1.5615E-02	1.67
1393769_at	---	1.5640E-02	1.10
1384098_at	Rnf125	1.5697E-02	1.36
1397213_at	---	1.5729E-02	1.13
1385344_at	---	1.5739E-02	1.64
1379765_at	Nlk	1.5757E-02	1.18
1377189_at	RGD1307390	1.5766E-02	1.86
1392597_at	RGD1305081	1.5796E-02	1.30
1373284_at	Sav1	1.5825E-02	1.25
1379234_a_at	Cdc45l	1.5834E-02	1.45
1385357_at	---	1.5840E-02	1.23
1371052_at	Nog	1.5886E-02	1.19
1371966_at	Pcmt1	1.5894E-02	1.27
1372523_at	Gclc	1.5973E-02	1.58
1370308_at	Xtp3tpa	1.5981E-02	1.60
1380398_at	---	1.5994E-02	1.74
1373023_at	---	1.6005E-02	1.21
1388890_at	---	1.6020E-02	1.15
1385723_at	---	1.6032E-02	1.33
1378472_at	---	1.6046E-02	2.91
1390542_at	Rad51c	1.6100E-02	1.33
1375979_at	---	1.6106E-02	1.29
1390220_at	---	1.6120E-02	1.19
1381511_at	---	1.6120E-02	1.59
1372539_at	---	1.6138E-02	3.63
1383344_at	---	1.6162E-02	1.45
1393057_at	LOC308320	1.6163E-02	1.19
1372541_at	Tmem138	1.6164E-02	1.34
1371740_at	Sike	1.6196E-02	1.18
1386985_at	Gstm1	1.6201E-02	1.45
1372628_at	Ap4s1	1.6201E-02	1.25
1377245_a_at	RGD1311723	1.6210E-02	1.55
1393793_at	---	1.6214E-02	1.94

1368825_at	Shox2	1.6217E-02	1.53
1389979_at	Tnpo3	1.6225E-02	1.24
1399035_at	Polr3h	1.6296E-02	1.24
1398938_at	Acp1	1.6298E-02	1.23
1394431_at	Cdadc1	1.6317E-02	1.14
1367716_at	Itfg1	1.6411E-02	1.20
1383286_at	Plek2	1.6421E-02	1.32
1377384_at	Plekhh3	1.6430E-02	1.43
1377533_at	Dmrtc1a	1.6439E-02	1.20
1388105_at	Cdc123	1.6449E-02	1.16
1379879_at	---	1.6481E-02	1.38
1379824_at	Tox	1.6499E-02	1.61
1396669_at	---	1.6502E-02	1.21
1376410_at	Mmp17	1.6513E-02	1.69
1383964_at	Ankrd50	1.6612E-02	1.23
1373629_at	Slc7a6	1.6654E-02	1.14
1383741_at	---	1.6685E-02	1.33
1390881_at	Abra	1.6736E-02	1.12
1391923_at	---	1.6758E-02	1.65
1398216_at	---	1.6779E-02	1.11
1384000_at	Sox4	1.6787E-02	2.80
1393082_at	Ppp1r14c	1.6822E-02	1.57
1372154_at	RGD1359616	1.6827E-02	1.09
1387515_at	Nmbr	1.6829E-02	1.26
1377037_at	Acot4	1.6867E-02	1.93
1372622_at	RGD1560778	1.6877E-02	1.81
1373722_at	Kif20a	1.6880E-02	1.72
1395661_at	---	1.6898E-02	1.13
1389896_at	---	1.6906E-02	1.19
1374359_at	Ccne2	1.6946E-02	1.24
1398473_at	Bloc1s2	1.6962E-02	1.28
1383958_at	Cdca2	1.6982E-02	1.39
1370325_at	Gorasp2	1.6988E-02	1.14
1379684_at	---	1.6989E-02	1.36
1385923_at	Id4	1.7004E-02	3.15
1380621_at	Fes	1.7005E-02	1.61
1379849_at	Thoc3	1.7028E-02	1.20
1390576_at	LOC684233	1.7051E-02	1.34
1373448_at	Acyp1	1.7101E-02	1.22
1376085_at	Tmem185a	1.7144E-02	1.23
1390231_at	---	1.7211E-02	1.85

1373930_at	LOC687681	1.7219E-02	1.21
1382065_at	---	1.7230E-02	1.43
1381927_at	LOC691223	1.7284E-02	1.36
1391255_at	Zmynd15	1.7284E-02	1.13
1382803_at	Map4k1	1.7315E-02	1.10
1397304_at	Igtp	1.7322E-02	1.80
1371899_at	Prkra	1.7393E-02	1.13
1392999_at	Neto2	1.7419E-02	1.43
1370806_at	Retsat	1.7440E-02	1.33
1382008_at	RGD1309804	1.7440E-02	1.23
1382783_at	Blm	1.7453E-02	1.57
1367929_at	Cd59	1.7454E-02	1.15
1389474_at	Mylip	1.7513E-02	1.34
1368933_at	Adarb1	1.7575E-02	1.78
1371905_at	MGC94190	1.7576E-02	1.25
1389181_at	---	1.7581E-02	1.39
1379356_at	---	1.7657E-02	1.72
1398341_at	Cisd3 /// LOC68	1.7686E-02	1.29
1386041_a_at	Klf2	1.7693E-02	2.47
1378334_a_at	---	1.7740E-02	1.29
1369956_at	Ifngr1	1.7752E-02	1.26
1382314_at	G1p2	1.7755E-02	1.38
1389646_at	Cdc23	1.7781E-02	1.17
1368465_at	Accn1	1.7794E-02	1.19
1374864_at	Spry2	1.7820E-02	1.99
1373169_at	Agpat5	1.7950E-02	1.28
1381327_a_at	Cr2	1.7958E-02	1.22
1388620_at	Tada3l	1.7962E-02	1.25
1386348_at	---	1.7969E-02	1.18
1391904_at	---	1.7994E-02	1.38
1382812_at	RGD1310429	1.8057E-02	1.23
1385239_at	---	1.8121E-02	1.11
1371814_at	Ube2g2	1.8124E-02	1.16
1371190_at	---	1.8125E-02	1.24
1388836_at	Prkch	1.8197E-02	1.23
1380872_at	---	1.8211E-02	1.24
1395182_at	Klhl20	1.8256E-02	1.23
1368402_at	Dync1li2	1.8267E-02	1.23
1397504_at	Wsb2	1.8279E-02	1.20
1380427_at	---	1.8320E-02	1.11
1376806_at	---	1.8326E-02	1.43

1381014_at	Ifi44	1.8348E-02	1.37
1385809_at	RGD1563839	1.8356E-02	1.20
1373907_at	Trappc4	1.8356E-02	1.19
1385761_s_at	---	1.8361E-02	1.19
1391878_at	RGD1560020_p	1.8388E-02	1.31
1378452_at	Prss36	1.8438E-02	1.25
1376374_at	---	1.8441E-02	1.14
1382682_at	---	1.8462E-02	1.44
1384288_at	---	1.8479E-02	1.28
1376898_at	---	1.8490E-02	1.10
1390621_at	RGD1308290	1.8495E-02	1.16
1372229_at	Pdk3	1.8507E-02	1.49
1368174_at	Egln3	1.8527E-02	4.41
1372294_at	Pxdn	1.8533E-02	1.71
1379411_at	RGD1563825	1.8534E-02	2.64
1387778_at	Sdf4	1.8579E-02	1.15
1381237_at	---	1.8621E-02	1.14
1384751_at	Rbm8	1.8660E-02	1.29
1367887_at	Lcat	1.8684E-02	1.36
1384130_at	RGD1560171	1.8743E-02	1.16
1392930_at	Armc1	1.8746E-02	1.23
1387927_a_at	Olfm1	1.8773E-02	1.59
1392998_at	---	1.8791E-02	1.93
1394577_at	---	1.8906E-02	1.51
1374840_at	RGD1564921	1.8962E-02	1.70
1372019_at	Med10	1.8982E-02	1.15
1367628_at	Lgals1	1.8993E-02	1.14
1380824_at	Hook3	1.9110E-02	1.48
1371308_at	Rps4x	1.9163E-02	1.04
1371537_at	B4galt5	1.9168E-02	1.80
1395438_at	---	1.9173E-02	1.45
1392240_at	---	1.9242E-02	1.39
1376980_at	Htr2c	1.9316E-02	1.09
1372269_at	Med6	1.9319E-02	1.12
1378316_at	---	1.9331E-02	1.39
1380079_at	RGD1561472	1.9446E-02	1.12
1375972_at	RGD1307493	1.9507E-02	1.53
1382469_at	FAM120C	1.9519E-02	1.20
1391830_at	Cpne8	1.9542E-02	1.34
1367491_at	Api5	1.9606E-02	1.34
1379597_at	---	1.9665E-02	1.37

1389359_at	Smc4	1.9669E-02	1.30
1391466_at	---	1.9681E-02	1.20
1372569_at	Fhl3	1.9769E-02	1.32
1390475_at	Rngtt	1.9822E-02	1.33
1384930_at	LOC687047	1.9854E-02	1.06
1380021_at	Dennd2d	1.9879E-02	1.49
1398872_at	Rps13	1.9898E-02	1.09
1376765_at	Mro	1.9904E-02	1.58
1384695_at	Upk3a	1.9914E-02	1.75
1392595_at	Znf618	1.9930E-02	1.52
1373830_at	LOC619574	2.0011E-02	1.30
1391719_at	---	2.0031E-02	1.24
1398707_at	---	2.0077E-02	1.63
1391518_at	LOC689399	2.0104E-02	1.42
1383925_at	Iip45	2.0122E-02	1.21
1391806_at	LOC498793	2.0138E-02	1.96
1372349_at	Uchl5ip	2.0261E-02	1.46
1371882_a_at	Sh2bpsm1	2.0280E-02	1.17
1372685_at	Cdkn3	2.0297E-02	1.27
1377182_at	Itgb3bp	2.0371E-02	1.32
1399062_at	---	2.0385E-02	1.13
1380303_at	LOC687827	2.0387E-02	1.31
1389858_at	Tk1	2.0405E-02	1.12
1385909_at	---	2.0420E-02	1.18
1368077_at	Fbp1	2.0434E-02	2.13
1371122_at	Tank	2.0469E-02	1.30
1378803_at	Nkx6-2	2.0476E-02	1.13
1377983_at	---	2.0496E-02	1.23
1371509_at	Tbrg1	2.0623E-02	1.26
1390615_at	Kpna1	2.0652E-02	1.27
1376921_at	---	2.0725E-02	1.05
1397881_at	Ccdc88a	2.0763E-02	1.25
1370974_at	Vps54	2.0789E-02	1.15
1372078_at	Strap	2.0796E-02	1.09
1388114_at	RGD1309537	2.0825E-02	1.36
1367976_at	Tpp2	2.0867E-02	1.27
1371036_at	Nrcam	2.0912E-02	1.33
1372815_at	Magoh	2.0923E-02	1.24
1367923_at	Acsbg1	2.0956E-02	1.15
1394940_at	Fam46a	2.1015E-02	1.76
1372670_at	RGD1307682	2.1019E-02	1.17

1377093_at	---	2.1050E-02	1.12
1372648_at	---	2.1078E-02	1.37
1388916_at	---	2.1088E-02	1.56
1392506_at	Cryzl1	2.1118E-02	1.23
1395255_at	---	2.1216E-02	1.25
1374659_at	Arpp-21	2.1219E-02	1.71
1392885_at	Mbd1	2.1229E-02	1.13
1378292_at	---	2.1253E-02	2.08
1390006_at	Rbm15b	2.1268E-02	1.18
1371395_at	Cbx3	2.1303E-02	1.13
1375300_at	Wnk1	2.1305E-02	1.35
1374932_at	---	2.1344E-02	2.71
1395368_at	---	2.1367E-02	1.42
1395757_at	---	2.1383E-02	1.27
1371611_at	Ext2	2.1421E-02	1.22
1368187_at	Gpnmb	2.1426E-02	1.05
1375900_at	Tnfrsf9	2.1437E-02	1.48
1388651_at	Rchy1	2.1439E-02	1.25
1378568_a_at	RGD1306228	2.1461E-02	1.37
1393231_at	Ppp4r2	2.1478E-02	1.40
1374752_at	Mdfic	2.1489E-02	1.85
1373490_at	Gmfg	2.1505E-02	1.79
1374376_at	Rgnef	2.1535E-02	1.39
1368338_at	Cd52	2.1576E-02	1.19
1392274_at	---	2.1642E-02	1.34
1398861_at	Nxf1	2.1651E-02	1.23
1375026_at	Calml4	2.1659E-02	1.22
1373577_at	Nrp1	2.1720E-02	1.29
1393817_at	---	2.1780E-02	1.29
1390224_at	---	2.1810E-02	1.15
1388323_at	Ndufa9	2.1829E-02	1.12
1392381_at	---	2.1834E-02	1.25
1372014_at	Med27	2.1839E-02	1.26
1390203_at	---	2.1845E-02	1.32
1384174_at	---	2.1866E-02	1.49
1372320_at	Msl3l1	2.1954E-02	1.18
1370827_at	Cyb5r4	2.1967E-02	1.22
1379547_at	---	2.1976E-02	1.15
1378076_at	---	2.2021E-02	1.51
1368571_at	Clip2	2.2094E-02	1.20
1384281_at	---	2.2096E-02	1.28

1379441_at	Znf294	2.2174E-02	1.18
1386571_at	RGD1562626	2.2201E-02	1.33
1381864_at	---	2.2225E-02	1.40
1390861_at	Zfp709l2	2.2255E-02	1.35
1397132_at	Zfp207	2.2257E-02	1.18
1382865_at	Tsga14	2.2257E-02	1.18
1373249_at	Ubl4	2.2262E-02	1.74
1374749_at	---	2.2269E-02	1.40
1372289_at	Slc25a12	2.2289E-02	1.11
1388703_at	Esam	2.2305E-02	1.07
1377968_at	---	2.2341E-02	1.57
1370645_at	Vom2r18	2.2373E-02	1.32
1376387_at	---	2.2392E-02	1.32
1378944_at	---	2.2440E-02	1.08
1388410_at	Ugp2	2.2515E-02	1.33
1373798_at	RGD1309139	2.2541E-02	1.19
1371467_at	LOC293103	2.2594E-02	1.19
1371024_at	Cux1	2.2594E-02	1.28
1370365_at	Gss	2.2641E-02	1.74
1392501_at	---	2.2643E-02	2.50
1379258_at	Klhl5	2.2688E-02	1.34
1385555_at	Fam101a	2.2697E-02	1.24
1376498_at	Rilpl1	2.2703E-02	1.45
1382794_at	---	2.2724E-02	1.40
1392191_at	LOC691093	2.2770E-02	1.32
1372261_at	---	2.2825E-02	1.39
1382251_at	Kpna1	2.2836E-02	1.41
1386310_at	---	2.2866E-02	1.18
1393087_at	Recql5	2.2902E-02	1.21
1372070_at	Ifi30	2.2912E-02	1.78
1373406_at	Tor1b	2.2928E-02	1.32
1373272_at	Plekha5	2.2974E-02	1.52
1378828_at	---	2.2995E-02	1.17
1385718_at	RGD1308031	2.3063E-02	1.26
1379285_at	Rtp4	2.3078E-02	1.50
1367803_at	Nup54	2.3079E-02	1.63
1394487_at	---	2.3126E-02	1.31
1383503_at	---	2.3144E-02	1.51
1393234_at	LOC687105	2.3260E-02	2.07
1382814_at	Odz3	2.3325E-02	1.69
1396205_at	---	2.3348E-02	1.27

1377202_at	Smc6l1	2.3376E-02	1.31
1388593_at	Mapk8ip3	2.3411E-02	1.72
1389497_at	Smyd5	2.3447E-02	1.45
1392873_at	---	2.3458E-02	1.10
1380933_at	---	2.3469E-02	1.26
1389444_at	Zdhhc20	2.3550E-02	1.59
1392111_at	Exosc3	2.3580E-02	1.50
1384615_at	Crop	2.3640E-02	1.22
1390338_at	LOC361646	2.3739E-02	1.51
1378396_at	---	2.3770E-02	1.33
1383736_at	Elavl2	2.3776E-02	1.52
1372643_at	Epb4.1l2	2.3780E-02	1.36
1367826_at	Nfe2l2	2.3785E-02	1.24
1387245_at	Lipf	2.3796E-02	1.10
1394209_at	---	2.3809E-02	1.23
1369206_at	Cpb2	2.3882E-02	1.25
1376693_at	RGD1563091	2.3894E-02	1.43
1375079_at	---	2.3959E-02	1.13
1398551_at	Ttc25	2.4006E-02	2.16
1381979_at	Sumf2	2.4019E-02	1.20
1369181_at	Cybb	2.4031E-02	1.19
1373876_at	Eif4e2	2.4088E-02	1.66
1388194_at	Dlat	2.4095E-02	1.62
1374912_at	Kif2c	2.4122E-02	1.35
1398866_at	Magi3	2.4205E-02	1.15
1380023_at	---	2.4254E-02	1.38
1389408_at	Rrm2	2.4272E-02	1.51
1389468_at	Rpia	2.4313E-02	1.45
1385132_at	---	2.4342E-02	1.57
1398612_at	Akr1c12	2.4451E-02	1.32
1370831_at	Mgll	2.4485E-02	2.49
1386754_at	Trim14	2.4500E-02	1.43
1388458_at	Rfc4	2.4506E-02	1.60
1369894_at	Htr2a	2.4628E-02	1.32
1372409_at	Mad2l1bp	2.4636E-02	1.35
1374544_at	MGC112715	2.4653E-02	1.10
1379023_at	---	2.4675E-02	1.33
1383950_at	---	2.4722E-02	1.20
1377026_a_at	---	2.4731E-02	1.11
1373186_at	Slain2	2.4741E-02	1.32
1398358_a_at	Itgb5	2.4759E-02	1.68

1392686_at	Nkd2	2.4822E-02	1.10
1372844_at	Efna1	2.4863E-02	1.88
1383572_at	Zdhhc6	2.4874E-02	1.11
1375899_at	---	2.4980E-02	1.22
1374758_at	---	2.5009E-02	1.33
1376569_at	Klf2	2.5025E-02	2.82
1373848_at	RGD1308584	2.5079E-02	1.21
1374285_at	---	2.5181E-02	1.34
1374935_at	---	2.5209E-02	1.34
1376485_at	Traip	2.5266E-02	1.47
1368267_at	Pomt1	2.5280E-02	1.11
1390188_at	Mrpl51	2.5280E-02	1.34
1391494_at	---	2.5335E-02	1.33
1384449_at	---	2.5351E-02	1.22
1376231_at	Gins1	2.5389E-02	1.55
1388583_at	Cxcl12	2.5425E-02	1.63
1386979_at	Tpo1	2.5435E-02	1.21
1367829_at	Echs1	2.5453E-02	1.14
1381151_at	---	2.5478E-02	1.28
1398777_at	Psmb6	2.5486E-02	1.21
1369930_at	Psma6	2.5575E-02	1.28
1373286_at	Fblim1	2.5579E-02	1.24
1373050_at	Tbc1d1	2.5600E-02	1.16
1377988_at	RGD1304694	2.5703E-02	1.12
1376112_a_at	Nutf2	2.5769E-02	1.19
1391711_at	---	2.5849E-02	1.46
1392040_at	Sass6	2.5859E-02	1.95
1396285_at	---	2.5871E-02	1.26
1369007_at	Nr4a2	2.5874E-02	1.32
1376192_at	Nat9	2.5998E-02	1.16
1372411_at	LOC684755	2.6007E-02	1.24
1390823_at	Skp2	2.6105E-02	1.51
1368209_at	Pdzk1ip1	2.6166E-02	1.83
1368145_at	Pcp4	2.6295E-02	1.33
1394511_at	Mysm1	2.6297E-02	1.28
1375244_at	---	2.6393E-02	1.11
1394419_at	Arhgap11a	2.6396E-02	1.32
1378952_at	Tmem101	2.6467E-02	1.26
1374052_at	Fam160b1	2.6514E-02	1.25
1370954_at	P4ha1	2.6632E-02	1.19
1374056_at	LOC690139	2.6636E-02	1.15

1378482_at	Mdfic	2.6636E-02	1.34
1389073_at	Reep4	2.6648E-02	1.14
1369852_at	F10	2.6750E-02	1.13
1389918_at	LOC290704	2.6914E-02	2.79
1383014_at	---	2.6942E-02	1.05
1383152_at	Chid1	2.6968E-02	1.25
1393383_at	Arhgdig	2.6980E-02	1.44
1388722_at	Dnajb1	2.6991E-02	1.27
1373334_at	Tmem129	2.6995E-02	1.18
1388443_at	Cdk2ap1 /// LO(	2.6997E-02	1.15
1383183_at	Dnm1l	2.7019E-02	1.33
1385088_at	RGD1310262	2.7112E-02	1.16
1381298_at	Mcm10	2.7151E-02	1.25
1391727_at	---	2.7193E-02	1.53
1391803_at	---	2.7197E-02	1.19
1377236_at	---	2.7391E-02	1.20
1382568_at	---	2.7425E-02	1.26
1369931_at	Pkm2	2.7527E-02	1.28
1391602_at	Drg1	2.7544E-02	1.39
1382419_at	Cenpk	2.7628E-02	1.64
1369546_at	Bbox1	2.7709E-02	1.14
1390889_at	LOC691543	2.7732E-02	1.32
1378723_at	---	2.7790E-02	1.24
1376116_at	Ddx24	2.7829E-02	1.19
1378874_at	Cdk2	2.7867E-02	1.24
1375073_at	RGD1310271	2.7906E-02	1.34
1368704_a_at	Cspg5	2.7918E-02	1.58
1373372_at	LOC501282	2.7942E-02	1.27
1373777_at	---	2.8005E-02	1.77
1388791_at	RGD1309930	2.8056E-02	1.35
1392385_at	Ncoa3	2.8060E-02	1.52
1379476_at	Nup35	2.8072E-02	1.25
1388927_at	Rabl4	2.8079E-02	1.25
1374620_at	Ceacam1	2.8148E-02	1.56
1393305_at	---	2.8161E-02	1.18
1375959_at	Nkd1	2.8171E-02	1.25
1373225_at	---	2.8195E-02	1.21
1373978_at	Ncbp1	2.8226E-02	1.33
1377004_at	---	2.8259E-02	1.13
1386890_at	S100a10	2.8307E-02	1.29
1380904_at	---	2.8309E-02	1.33

1368902_at	Pak3	2.8365E-02	1.55
1398458_at	RGD1307284	2.8432E-02	1.51
1376701_a_at	MGC94282	2.8504E-02	1.21
1370299_at	Aldob	2.8530E-02	1.35
1378507_at	---	2.8584E-02	1.13
1375183_at	Id4	2.8663E-02	2.50
1390662_at	Trim24	2.8670E-02	1.56
1391026_at	Ier5l	2.8682E-02	1.20
1393799_at	Unc5b	2.8730E-02	1.21
1382120_at	Epc2	2.8836E-02	1.19
1372515_at	---	2.8865E-02	1.55
1370851_a_at	Kalrn	2.8951E-02	1.34
1388590_at	Znrd1	2.8954E-02	1.19
1371463_at	Phf5a	2.9065E-02	1.25
1395837_at	---	2.9074E-02	1.13
1371901_at	---	2.9080E-02	1.65
1377042_at	Pcgf5	2.9240E-02	1.19
1371320_at	Itm2b	2.9292E-02	1.42
1372490_at	---	2.9317E-02	1.46
1389955_at	---	2.9379E-02	1.29
1368107_at	Prl3c1	2.9413E-02	1.07
1389603_at	---	2.9445E-02	1.31
1370875_at	Ezr	2.9465E-02	1.27
1391063_at	Kif23	2.9485E-02	1.53
1384338_at	Cdc45l	2.9507E-02	1.31
1389220_at	LOC682679	2.9512E-02	1.39
1391461_at	RGD1306576	2.9519E-02	1.14
1389001_at	---	2.9520E-02	1.21
1370305_at	Yif1	2.9526E-02	1.10
1377661_at	Frs2	2.9583E-02	1.17
1384842_s_at	Fas	2.9598E-02	1.60
1395039_at	---	2.9695E-02	1.62
1392349_at	Slc5a3	2.9703E-02	1.20
1370829_at	Fntb	2.9760E-02	1.24
1379526_at	---	2.9784E-02	1.27
1390456_at	---	2.9799E-02	1.36
1374347_at	Cdkal1	2.9829E-02	1.18
1391947_at	---	2.9832E-02	1.15
1382310_at	RGD1563296	2.9891E-02	1.30
1373638_at	---	3.0013E-02	1.22
1379825_at	---	3.0045E-02	1.33

1392909_at	---	3.0047E-02	1.50
1383423_at	---	3.0073E-02	1.08
1388448_at	Cdc42se2 /// LC	3.0105E-02	1.11
1388456_at	S100a1	3.0145E-02	1.22
1378175_at	---	3.0199E-02	1.29
1376521_at	---	3.0207E-02	1.14
1388972_at	Rtn4r	3.0211E-02	1.29
1389710_at	Sos1	3.0252E-02	1.23
1393397_at	Cpa2	3.0261E-02	1.33
1376654_at	RGD1308448	3.0301E-02	2.48
1388100_at	Cdk5rap2	3.0316E-02	1.36
1393892_at	RGD1306625	3.0378E-02	1.64
1376039_at	Aurka	3.0383E-02	1.55
1391631_at	---	3.0394E-02	1.45
1381729_at	LOC498675	3.0421E-02	1.28
1393158_at	Cenpq	3.0426E-02	1.55
1374540_at	Cdca7	3.0430E-02	1.23
1383289_at	Nif3l1	3.0444E-02	1.17
1385151_at	---	3.0493E-02	1.45
1373339_at	Letmd1	3.0495E-02	1.07
1383134_at	RGD1311612	3.0497E-02	1.19
1383745_at	---	3.0502E-02	1.13
1384150_at	Mid1	3.0502E-02	1.73
1380665_at	---	3.0569E-02	1.24
1397198_at	---	3.0582E-02	1.05
1371266_at	Afm	3.0607E-02	1.43
1373465_at	Pqlc1	3.0619E-02	1.17
1397704_at	LOC691979	3.0647E-02	1.32
1376599_at	Atad2	3.0654E-02	1.27
1395131_at	---	3.0655E-02	1.10
1380130_at	---	3.0698E-02	1.41
1392165_at	Ing2	3.0700E-02	1.41
1369973_at	Xdh	3.0727E-02	1.12
1368051_at	Hsd17b12	3.0737E-02	1.34
1389374_at	Kifc3	3.0829E-02	1.27
1390580_at	---	3.0839E-02	1.26
1384561_at	RGD1561983	3.0905E-02	1.21
1370025_at	Pip4k2c	3.0936E-02	1.13
1397266_at	---	3.1019E-02	1.76
1387676_at	Mark1	3.1027E-02	1.25
1379422_at	Alg13	3.1045E-02	1.11

1393844_at	Mlf1ip	3.1177E-02	1.38
1371415_at	Uqcrh	3.1205E-02	1.10
1369008_a_at	Olfm1	3.1206E-02	1.47
1375934_at	Rnf128	3.1208E-02	1.46
1390034_at	Ralgps2	3.1278E-02	1.87
1378875_a_at	RGD1303066	3.1314E-02	1.43
1374139_at	Cdr2	3.1377E-02	1.65
1380148_at	---	3.1400E-02	1.29
1395945_at	---	3.1400E-02	1.28
1372681_at	---	3.1403E-02	1.61
1383265_at	Tbc1d23	3.1476E-02	1.21
1399121_at	---	3.1555E-02	1.13
1387798_a_at	Cr1l	3.1620E-02	1.21
1383684_at	Asf1b	3.1640E-02	1.42
1389720_at	---	3.1664E-02	1.61
1370781_a_at	Kcnip1	3.1748E-02	1.18
1383742_at	Snx7	3.1760E-02	1.18
1370007_at	Pdia4	3.1790E-02	1.71
1379463_at	Filip1	3.1826E-02	2.17
1375794_at	---	3.2017E-02	1.13
1393751_at	Fabp12	3.2093E-02	1.07
1390489_at	Syncrip	3.2100E-02	1.17
1367694_at	Hadhb	3.2148E-02	1.34
1379774_at	---	3.2150E-02	1.21
1383977_a_at	---	3.2190E-02	1.18
1386474_at	---	3.2249E-02	1.22
1396014_at	Timm8a2	3.2255E-02	1.23
1376609_at	RGD1309102	3.2262E-02	1.07
1390305_at	Prkcbp1	3.2373E-02	1.22
1368376_at	Nr0b2	3.2426E-02	1.51
1387825_at	Ugt2b	3.2469E-02	1.15
1384828_at	Kif7	3.2546E-02	1.33
1374802_at	RGD1305288	3.2547E-02	1.18
1390646_at	---	3.2575E-02	1.20
1386466_at	---	3.2606E-02	1.54
1368136_at	Tmpo	3.2649E-02	1.32
1380198_at	---	3.2659E-02	1.20
1392377_at	---	3.2670E-02	1.10
1370910_at	Rfc2	3.2761E-02	1.27
1382756_at	Kpna1	3.2773E-02	1.21
1382937_at	Spdef	3.2776E-02	1.22

1387008_at	Sfxn3	3.2836E-02	1.22
1389566_at	Ccnb2	3.2855E-02	1.45
1374031_at	Parp6	3.2931E-02	1.19
1374177_at	Taf13	3.2971E-02	1.25
1372396_at	RGD1308026	3.2984E-02	1.22
1393036_at	Psrc1	3.2994E-02	1.43
1367847_at	Nupr1	3.3081E-02	1.92
1383247_a_at	rCG_35099	3.3088E-02	1.32
1380953_at	---	3.3112E-02	1.14
1378968_at	Bcl2l14	3.3127E-02	1.16
1374273_at	---	3.3130E-02	1.34
1370522_at	Gcgr	3.3220E-02	1.55
1389734_x_at	H2-T24	3.3233E-02	1.55
1390623_at	Gins3	3.3281E-02	1.22
1371229_at	Atpif1	3.3323E-02	1.20
1372755_at	Mal2	3.3327E-02	1.32
1392131_at	---	3.3401E-02	1.33
1371414_at	Gsn	3.3455E-02	1.11
1375904_at	---	3.3458E-02	1.27
1390501_at	---	3.3478E-02	1.10
1380143_at	---	3.3483E-02	1.16
1398380_at	Vwa1	3.3576E-02	1.23
1391084_at	---	3.3583E-02	1.12
1386774_at	Fam5b	3.3613E-02	1.84
1399009_at	Ints4	3.3621E-02	1.23
1382773_at	---	3.3665E-02	1.19
1370685_at	Trpv1	3.3671E-02	1.21
1391889_at	---	3.3712E-02	1.31
1393391_at	Tgs1	3.3786E-02	1.20
1398665_at	---	3.3795E-02	1.28
1379200_at	RGD1563278	3.3832E-02	1.25
1371319_at	Itm2b	3.3859E-02	1.26
1381777_at	---	3.3909E-02	1.44
1398439_a_at	Orc6l	3.3987E-02	1.55
1373283_at	---	3.4027E-02	1.20
1379205_at	---	3.4109E-02	1.27
1370499_at	Klrb1a	3.4223E-02	1.13
1375430_at	RGD1311260	3.4255E-02	1.22
1376138_at	---	3.4258E-02	1.85
1376011_at	---	3.4307E-02	1.58
1380107_a_at	Nat8l	3.4331E-02	1.10

1379525_at	Crls1	3.4347E-02	1.22
1386162_at	Txk	3.4354E-02	1.40
1376607_a_at	RGD1310414	3.4443E-02	1.30
1376983_at	LOC680200	3.4452E-02	1.23
1387059_at	Stk39	3.4452E-02	1.33
1391069_at	RGD1309748	3.4482E-02	1.25
1381995_at	Brunol4	3.4521E-02	1.07
1376886_at	---	3.4626E-02	1.27
1369071_at	S1pr1	3.4693E-02	1.15
1377380_at	---	3.4712E-02	1.37
1384430_at	Dph3	3.4723E-02	1.19
1390684_at	---	3.4732E-02	1.38
1369934_at	Ppib	3.4777E-02	1.18
1373583_at	---	3.4801E-02	1.27
1384587_at	---	3.4835E-02	1.59
1379982_at	---	3.4860E-02	1.23
1383845_at	---	3.4867E-02	1.17
1397872_at	---	3.4929E-02	1.65
1389387_at	rCG_37297	3.4935E-02	1.15
1398087_at	---	3.4951E-02	1.11
1372734_at	Smagp	3.5017E-02	1.55
1379368_at	Bcl6	3.5059E-02	1.23
1376652_at	C1qa	3.5086E-02	1.38
1390025_at	Rp9	3.5129E-02	1.32
1393698_at	Dido1	3.5189E-02	1.23
1368914_at	Runx1	3.5225E-02	1.34
1372152_at	Alg14	3.5250E-02	1.23
1380870_at	---	3.5302E-02	1.19
1396314_at	---	3.5326E-02	1.12
1376319_at	Sema3c	3.5341E-02	1.41
1389509_at	Sap130	3.5366E-02	1.13
1367502_at	Mrpl21	3.5383E-02	1.14
1380141_at	Nsun4	3.5392E-02	1.13
1381252_at	---	3.5425E-02	1.29
1389751_at	---	3.5466E-02	1.15
1391152_at	LOC367381 /// I	3.5468E-02	1.24
1374831_at	Athl1	3.5479E-02	1.20
1392272_at	LOC682772	3.5512E-02	1.22
1391119_at	---	3.5520E-02	1.15
1391749_a_at	Nxnl2	3.5601E-02	1.48
1378483_at	Prl7b1	3.5642E-02	1.37

1388523_at	Txndc12	3.5646E-02	1.38
1392543_at	LOC503237 /// I	3.5758E-02	1.09
1396292_at	---	3.5791E-02	1.32
1367836_at	Cpt1a	3.5895E-02	1.92
1378045_at	C1ql1	3.5912E-02	1.11
1397746_at	---	3.5983E-02	1.34
1399041_at	Opa3	3.6004E-02	1.27
1393723_at	RGD1305038	3.6040E-02	1.63
1385749_at	---	3.6043E-02	1.21
1380700_at	Arhgap27	3.6070E-02	1.29
1380572_at	---	3.6079E-02	1.16
1392798_at	Arhgdig /// Pdia	3.6083E-02	1.97
1395578_at	---	3.6144E-02	1.29
1396545_at	---	3.6211E-02	1.27
1383258_at	RGD1310587	3.6273E-02	1.37
1374014_at	Nsmaf	3.6275E-02	1.24
1383960_at	Pex16	3.6316E-02	1.27
1378960_at	---	3.6516E-02	1.98
1377601_at	RGD1311257	3.6528E-02	1.40
1396328_at	---	3.6749E-02	1.22
1372359_at	Dusp26	3.6812E-02	1.31
1377967_at	Cdt1	3.6832E-02	1.22
1385411_at	rCG_32844	3.6841E-02	1.53
1388887_at	Ggta1	3.6916E-02	1.73
1369968_at	Ptn	3.6970E-02	1.32
1377982_at	Dtx4	3.7012E-02	1.19
1374747_at	Pftk1	3.7017E-02	1.37
1389485_at	Mdm1	3.7019E-02	1.10
1377925_at	---	3.7028E-02	1.10
1369663_at	Ephx2	3.7129E-02	1.36
1388505_at	---	3.7190E-02	1.20
1383581_at	LOC680531	3.7223E-02	1.29
1368347_at	Col5a3	3.7321E-02	2.42
1387551_at	Kcnh7	3.7321E-02	1.14
1376611_at	Pola1	3.7342E-02	1.36
1371210_s_at	RT1-Aw2 /// RT:	3.7498E-02	1.38
1397473_at	Dtnbp1	3.7571E-02	1.14
1390126_at	---	3.7603E-02	1.64
1375981_a_at	Suv39h1	3.7625E-02	1.34
1388392_at	Tax1bp3	3.7639E-02	1.26
1376690_at	Med21	3.7677E-02	1.34

1375554_at	Ddx47	3.7735E-02	1.14
1387048_at	Ddx39	3.7738E-02	1.21
1381278_at	---	3.7742E-02	1.61
1384570_at	Plekha4	3.7758E-02	1.15
1390525_a_at	Stra6	3.7835E-02	1.34
1389657_at	Znf524	3.7926E-02	1.06
1392864_at	Arhgap5	3.7937E-02	1.41
1395797_at	---	3.7948E-02	1.32
1383230_at	RGD1311078	3.8012E-02	1.08
1372602_at	Stbd1	3.8074E-02	1.59
1367768_at	Lxn	3.8161E-02	1.69
1377158_at	---	3.8201E-02	1.28
1368923_at	Ecel1	3.8226E-02	1.26
1383290_at	Spint1	3.8237E-02	1.37
1389369_at	---	3.8291E-02	1.25
1384394_at	---	3.8408E-02	1.82
1377389_at	---	3.8416E-02	1.44
1387670_at	Gpd2	3.8417E-02	2.11
1384488_at	Cdkn2d	3.8535E-02	1.25
1387788_at	Junb	3.8537E-02	1.10
1376685_at	---	3.8543E-02	1.87
1394330_at	---	3.8579E-02	1.37
1376133_at	Mdp-1	3.8591E-02	1.34
1393662_at	---	3.8609E-02	1.20
1391320_at	---	3.8616E-02	1.12
1384996_at	---	3.8678E-02	1.29
1388019_at	Odf2	3.8718E-02	1.31
1367744_at	Maged2	3.8719E-02	1.35
1395256_at	---	3.8770E-02	1.10
1376470_at	Fnbp4	3.8789E-02	1.25
1371511_at	Arpc2	3.8822E-02	1.16
1390189_at	LOC298977	3.8905E-02	1.29
1376537_at	Ptpn3	3.9005E-02	1.27
1393828_at	Chid1	3.9016E-02	1.14
1369131_at	Slc18a2	3.9037E-02	1.28
1371636_at	Ankrd13c	3.9042E-02	1.18
1381994_at	---	3.9198E-02	1.41
1392957_at	Ssbp2	3.9208E-02	1.64
1374893_at	---	3.9240E-02	1.13
1383439_at	Npas2	3.9273E-02	1.35
1383768_at	Elavl2	3.9311E-02	1.44

1390495_at	---	3.9431E-02	1.68
1372880_at	Zbtb17	3.9488E-02	1.28
1372059_at	RGD1309437	3.9492E-02	1.57
1391022_at	Lamb3	3.9512E-02	1.68
1384862_at	---	3.9558E-02	1.69
1388425_at	Oaf	3.9575E-02	1.21
1382472_at	---	3.9597E-02	1.44
1378620_at	---	3.9602E-02	1.22
1392477_at	Etv1	3.9619E-02	1.10
1389556_at	Kifap3	3.9629E-02	1.17
1383926_at	Bub1b	3.9798E-02	1.28
1377544_at	Gpr68	3.9825E-02	1.07
1367476_at	Srp14	3.9895E-02	1.09
1386641_at	---	3.9910E-02	1.60
1387055_at	Nae1	3.9962E-02	1.06
1370870_at	Me1	4.0127E-02	1.25
1388319_at	---	4.0183E-02	1.16
1373996_at	---	4.0238E-02	1.34
1391227_at	---	4.0242E-02	1.62
1392089_at	Hcfc2	4.0297E-02	1.16
1382660_at	RGD1563084	4.0364E-02	1.27
1376095_at	---	4.0426E-02	1.41
1378294_at	Fbxl12	4.0434E-02	1.11
1379856_at	Arid1b /// LOC4	4.0465E-02	1.10
1377768_at	Kctd1	4.0517E-02	1.22
1391808_at	---	4.0530E-02	1.30
1395474_at	---	4.0597E-02	1.13
1373346_at	RGD1306508	4.0604E-02	1.21
1395139_at	Kif16b	4.0682E-02	1.19
1370980_at	Sftpb	4.0728E-02	1.09
1370907_at	St6gal1	4.0756E-02	1.42
1398486_at	LOC683007	4.0879E-02	1.82
1392566_at	---	4.0894E-02	1.05
1395523_at	Rbmx	4.0897E-02	1.39
1392512_at	Hist3h2ba	4.0923E-02	1.12
1368199_at	Nup88	4.0936E-02	1.33
1380461_at	---	4.0961E-02	1.41
1383665_at	Lpin2	4.0993E-02	1.53
1371883_at	Mmd	4.1012E-02	1.33
1393405_at	Xrcc2	4.1036E-02	1.19
1383273_a_at	Pcbp3	4.1104E-02	1.70

1374539_at	Atp10d	4.1248E-02	1.48
1380446_at	Mllt10	4.1277E-02	1.14
1368248_at	Cds1	4.1296E-02	1.22
1381935_at	Sspn	4.1321E-02	1.19
1378106_at	Phlda2	4.1342E-02	1.60
1390967_a_at	Cpsf3l	4.1357E-02	1.25
1390921_at	---	4.1376E-02	1.15
1380936_at	RGD1561665	4.1423E-02	1.15
1367662_at	Hsd17b10	4.1456E-02	1.26
1368622_at	Fbp2	4.1502E-02	1.62
1389506_x_at	Cdc20	4.1569E-02	1.37
1372128_at	Mrps12	4.1589E-02	1.13
1369902_at	Bmf	4.1650E-02	1.35
1391738_at	---	4.1657E-02	1.19
1368450_at	Myo5a	4.1710E-02	1.56
1386174_at	---	4.1756E-02	1.25
1367606_at	Rps3a	4.1778E-02	1.03
1389070_at	Trim33	4.1827E-02	1.17
1378930_a_at	---	4.1832E-02	1.62
1388256_at	RT1-A3	4.1851E-02	1.33
1367588_a_at	Rpl13a	4.1873E-02	1.11
1367578_at	Prdx2	4.1963E-02	1.18
1379264_at	Znrf1	4.1989E-02	1.44
1389555_at	Tcf19	4.2005E-02	1.30
1383729_at	LOC303566	4.2023E-02	1.36
1389616_at	---	4.2044E-02	1.37
1377628_at	---	4.2118E-02	1.17
1374635_at	LOC689663	4.2195E-02	1.33
1383458_at	---	4.2225E-02	1.41
1394636_at	---	4.2296E-02	1.26
1372886_at	Tacc3	4.2343E-02	1.53
1396078_at	Krit1	4.2358E-02	1.15
1385157_at	Cbx1	4.2476E-02	1.19
1384162_at	Csrnp2	4.2494E-02	1.31
1390127_at	Dixdc1	4.2540E-02	1.38
1384892_at	---	4.2598E-02	1.45
1391316_at	---	4.2694E-02	1.21
1380301_at	---	4.2720E-02	1.26
1393143_at	---	4.2728E-02	1.37
1382913_at	Cttnbp2	4.2730E-02	2.21
1392550_at	Dact1	4.2778E-02	1.14

1390282_at	Cyp2s1	4.2814E-02	1.40
1393091_at	Mier3	4.2847E-02	1.29
1388644_at	Mgll	4.2850E-02	2.12
1393275_at	---	4.2878E-02	1.07
1379066_at	Slc5a3	4.2905E-02	1.10
1387258_a_at	Pcmt1	4.2905E-02	1.16
1388867_at	MGC112830	4.2906E-02	1.13
1368368_a_at	Lsr	4.2942E-02	1.19
1393830_at	Sh2d2a	4.2958E-02	1.19
1398141_at	Srcrb4d	4.2964E-02	1.27
1371923_at	Lpcat1	4.2981E-02	1.39
1374613_at	---	4.2994E-02	1.54
1372765_a_at	Peci /// RGD131	4.3015E-02	2.04
1393395_at	Zmym4	4.3020E-02	1.15
1388479_at	---	4.3048E-02	1.50
1373964_at	LOC100174910	4.3085E-02	1.32
1374711_at	Cpsf3	4.3109E-02	1.19
1390553_at	Tmem194b	4.3115E-02	1.27
1384310_at	---	4.3118E-02	1.25
1392544_at	Rqcd1	4.3136E-02	1.27
1372862_at	Rab22a	4.3210E-02	1.10
1393906_at	Fam149a	4.3380E-02	1.15
1380448_at	Alkbh	4.3464E-02	1.32
1380887_at	---	4.3574E-02	1.28
1393487_at	Cenpa	4.3576E-02	1.10
1374844_at	---	4.3579E-02	1.20
1395336_at	RGD1309930	4.3594E-02	1.32
1391840_at	RGD1310693	4.3641E-02	1.21
1392057_at	LOC500893	4.3655E-02	1.30
1385541_at	Hsp90aa1 /// LC	4.3673E-02	1.16
1367575_at	Eno1	4.3746E-02	1.56
1393583_at	Etaa1	4.3769E-02	1.25
1398377_at	Znf672	4.3820E-02	1.12
1370408_at	Nid67	4.3828E-02	1.63
1392877_at	---	4.3831E-02	1.14
1374233_at	Fam45a	4.3832E-02	1.12
1372111_at	Cav1	4.3856E-02	1.63
1382727_at	---	4.3894E-02	1.13
1371445_at	Lrrc59	4.3914E-02	1.18
1378136_at	Lrp3	4.3939E-02	1.11
1383355_at	Abca1	4.4047E-02	1.29

1371428_at	Sec61g	4.4123E-02	1.08
1375954_at	S100a13	4.4132E-02	1.34
1389913_at	Lrrfip1	4.4139E-02	1.70
1385414_at	Cd8a	4.4149E-02	1.12
1398704_at	---	4.4191E-02	1.10
1369939_at	Cycs	4.4206E-02	1.09
1380425_at	Rnasel	4.4244E-02	1.29
1370171_at	Hnrnpu	4.4301E-02	1.11
1378369_at	Dact1	4.4306E-02	1.34
1387964_a_at	Ero1l	4.4342E-02	1.20
1389332_at	---	4.4349E-02	1.35
1392535_at	Pycrl	4.4361E-02	1.13
1398268_at	Nfyc	4.4452E-02	1.22
1378020_at	---	4.4522E-02	1.24
1369467_a_at	Pfkfb1	4.4599E-02	1.23
1386864_at	Pgam1	4.4624E-02	1.08
1376381_at	Xkr5	4.4632E-02	1.58
1386940_at	Timp2	4.4772E-02	1.39
1389182_at	RGD1311269	4.4777E-02	1.15
1389160_at	Eraf	4.4781E-02	1.32
1380110_at	Jak2	4.4821E-02	1.18
1382962_at	Lpar2	4.4837E-02	1.11
1386863_at	Ppp1ca	4.4856E-02	1.16
1382017_at	---	4.5012E-02	1.32
1387061_at	Jup	4.5028E-02	1.24
1388436_at	Snrpa	4.5121E-02	1.15
1370194_at	Snap23	4.5123E-02	1.20
1389989_at	Atrx	4.5155E-02	1.25
1367574_at	Vim	4.5214E-02	1.41
1398945_at	---	4.5217E-02	1.08
1368321_at	Egr1	4.5309E-02	1.34
1388484_at	Ube2c	4.5317E-02	1.47
1367971_at	Ptp4a2	4.5443E-02	1.22
1397542_at	---	4.5486E-02	2.13
1397598_at	---	4.5512E-02	1.13
1383843_at	Hlcs	4.5550E-02	1.26
1381524_at	---	4.5602E-02	1.17
1367494_at	RGD1310899	4.5621E-02	1.19
1391100_at	---	4.5654E-02	1.17
1373258_at	Ctsf	4.5718E-02	1.19
1382603_at	---	4.5752E-02	1.38

1371987_at	Pols	4.5761E-02	1.07
1383724_at	Hs2st1	4.5782E-02	1.29
1392463_at	Map7d2	4.5804E-02	1.34
1381244_at	---	4.5834E-02	1.56
1372072_at	Hdhd2	4.5848E-02	1.13
1390785_at	Ubxd4	4.5928E-02	1.28
1385990_at	RGD1565545	4.5966E-02	1.16
1368323_at	Tfpi	4.6107E-02	1.22
1389263_at	Rai14	4.6117E-02	1.32
1368725_at	Jag1	4.6137E-02	1.31
1396117_at	RGD1563838	4.6142E-02	1.29
1374312_at	Uck1	4.6151E-02	1.14
1376812_at	---	4.6264E-02	1.23
1368947_at	Gadd45a	4.6264E-02	1.16
1372687_at	Crct1	4.6356E-02	1.67
1376185_at	Kifc1	4.6357E-02	1.42
1385105_at	Ints7	4.6379E-02	1.29
1371334_at	Itm2c	4.6443E-02	1.29
1389184_at	Rpp30	4.6464E-02	1.43
1378790_at	---	4.6478E-02	1.21
1385098_at	---	4.6611E-02	1.30
1370095_at	Ltb4r	4.6615E-02	1.17
1373592_at	Serpinb9	4.6671E-02	1.16
1385360_at	---	4.6710E-02	1.28
1388488_at	Lsm3	4.6821E-02	1.42
1379619_at	---	4.6882E-02	1.70
1378129_a_at	---	4.6954E-02	1.20
1371388_at	Pdhb	4.6975E-02	1.48
1381858_at	Trim14	4.7005E-02	1.25
1381980_at	---	4.7015E-02	1.11
1368593_at	Cd1d1	4.7061E-02	1.16
1381400_at	---	4.7120E-02	1.39
1374262_at	---	4.7191E-02	1.52
1389481_at	RGD735065	4.7261E-02	1.15
1395276_at	Rnf7	4.7317E-02	1.12
1389810_at	Skiv2l2	4.7363E-02	1.22
1380429_at	---	4.7425E-02	1.22
1368888_a_at	Rtn4	4.7463E-02	1.08
1383536_at	Dhfr	4.7495E-02	1.34
1372156_at	Tmem97	4.7501E-02	1.25
1399088_at	Tlk2	4.7515E-02	1.16

1388752_at	Bclaf1	4.7542E-02	1.19
1368758_a_at	Galr2	4.7545E-02	1.24
1375862_at	Pxdn	4.7594E-02	1.28
1375711_at	---	4.7601E-02	1.25
1389460_at	---	4.7664E-02	1.36
1387024_at	Dusp6	4.7672E-02	1.95
1370127_at	Pold1	4.7749E-02	1.35
1370040_at	Ogg1	4.7849E-02	1.20
1389859_at	Csnk1a1	4.7872E-02	1.46
1397063_at	---	4.7874E-02	1.13
1374740_at	LOC688966	4.7916E-02	1.21
1378925_at	Crem	4.7952E-02	1.51
1373631_at	Rap1gap	4.8071E-02	1.41
1383750_at	---	4.8137E-02	1.21
1374474_at	Cpne8	4.8143E-02	1.50
1373290_at	Ezh2	4.8149E-02	1.48
1390561_at	---	4.8176E-02	1.22
1369158_at	Casr	4.8241E-02	1.62
1370281_at	Fabp5	4.8313E-02	1.37
1378033_at	---	4.8317E-02	1.32
1387955_at	Ugt2b5	4.8351E-02	1.24
1393574_at	---	4.8461E-02	1.32
1391735_at	---	4.8496E-02	1.75
1373389_at	Acad9	4.8595E-02	1.22
1375478_at	Zfp362 /// Zscar	4.8636E-02	1.07
1397642_at	Rad50	4.8668E-02	1.27
1384706_at	Peli2	4.8678E-02	1.24
1379633_a_at	Uba7	4.8691E-02	1.39
1372187_at	Prkd3	4.8731E-02	1.31
1375915_at	Irak1bp1	4.8789E-02	1.28
1388979_at	Smndc1	4.8860E-02	1.17
1384420_at	---	4.8864E-02	1.11
1379491_at	Mrap	4.8902E-02	1.24
1393816_at	LOC689399	4.8914E-02	1.23
1382095_at	---	4.8980E-02	1.30
1373858_at	---	4.8994E-02	1.10
1371133_a_at	Prkar2b	4.9057E-02	1.32
1389210_at	Lcp1	4.9090E-02	1.69
1374293_at	H2afj	4.9105E-02	1.33
1378264_at	Nasp	4.9122E-02	1.39
1369978_at	Prpsap2	4.9215E-02	1.10

1384661_at	RGD1565551	4.9253E-02	1.13
1379104_at	---	4.9258E-02	1.33
1375093_at	Rbks	4.9292E-02	1.40
1384604_at	---	4.9296E-02	1.18
1397972_at	---	4.9332E-02	1.25
1385379_at	Znf287	4.9350E-02	1.24
1390124_at	Fam98b	4.9419E-02	1.18
1370294_a_at	Cdc20	4.9457E-02	1.44
1393877_at	---	4.9458E-02	1.13
1395606_at	---	4.9494E-02	1.28
1388723_at	Bre	4.9515E-02	1.17
1377824_a_at	Igf2bp3	4.9519E-02	1.09
1381551_at	Alkbh	4.9523E-02	1.18
1367828_at	Acads	4.9550E-02	1.27
1384093_at	C1ql3	4.9700E-02	1.36
1382907_at	LOC310721	4.9725E-02	1.35
1396479_at	Zbtb7c	4.9841E-02	1.04
1389176_at	Inpp5f	4.9842E-02	1.27
1377595_at	Ssfa2	4.9853E-02	1.75
1388393_at	Plp2	4.9861E-02	1.14
1395476_at	---	4.9933E-02	1.20

Affy ID	Gene Symbol	Corr p-value	LOG Fold-Change
1394146_at	---	5.6700E-05	-1.17
1390986_at	---	6.9200E-05	-1.31
1387143_at	Ppp1r9b	1.6152E-04	-1.22
1390543_at	---	1.9400E-04	-1.59
1391600_at	Mga	1.9785E-04	-1.51
1383457_at	---	1.9961E-04	-1.33
1381441_at	LOC687813	2.6765E-04	-1.35
1369743_a_at	P2rx4	2.8849E-04	-1.35
1395630_at	Adcy9	3.4727E-04	-1.50
1390997_at	---	4.3909E-04	-1.32
1396178_at	---	5.0304E-04	-1.17
1374966_at	Dcx	5.1272E-04	-1.33
1380556_at	Fbxo10	7.9256E-04	-1.33
1377331_at	---	8.3061E-04	-1.19
1391269_at	---	8.8969E-04	-1.78
1373607_at	St3gal3	9.6493E-04	-1.34
1389832_at	Gsto1	1.0407E-03	-1.07
1388500_at	---	1.0587E-03	-1.73
1393109_at	---	1.1628E-03	-1.46
1369000_at	Ntrk1	1.2199E-03	-2.65
1379252_at	---	1.2262E-03	-1.45
1386904_a_at	Cyb5a	1.3300E-03	-1.57
1388952_at	RGD1307935	1.3317E-03	-1.12
1397971_at	---	1.3340E-03	-1.15
1383311_at	Mtmr11	1.3361E-03	-1.10
1381427_at	rCG_44068	1.3483E-03	-1.29
1390522_at	Fbxl11	1.4006E-03	-1.39
1368278_at	Lgals2	1.4031E-03	-1.57
1367571_a_at	Igf2	1.4216E-03	-1.45
1387826_at	Pdxk /// RGD156	1.5285E-03	-1.26
1374482_at	Ctps2	1.5435E-03	-1.12
1392513_at	RGD1307041	1.5640E-03	-1.13
1383064_at	---	1.6234E-03	-1.27
1369135_at	Syt11	1.6505E-03	-1.77
1376117_at	Slc44a4	1.7038E-03	-1.23
1373636_at	Spock1	1.7170E-03	-1.94
1385139_at	LOC500118	1.8199E-03	-1.24
1385474_at	Znf294	1.9081E-03	-1.14
1392430_at	---	1.9905E-03	-1.33
1398073_at	---	1.9907E-03	-1.12

1367721_at	Sdc4	2.0588E-03	-1.21
1368068_a_at	Pacsin2	2.2594E-03	-1.47
1390292_at	Tmem8	2.3329E-03	-1.32
1371651_at	Vps25	2.3466E-03	-1.24
1393699_at	---	2.4171E-03	-1.21
1379840_at	---	2.4917E-03	-1.15
1369431_at	Galnt7	2.6058E-03	-1.56
1372277_at	LOC641316	2.8924E-03	-1.73
1391818_at	Tmco4	2.9010E-03	-1.16
1370920_at	Srpk2	2.9888E-03	-1.14
1374112_at	Mapk1ip1	3.1262E-03	-1.30
1390169_at	LOC688749	3.1415E-03	-1.25
1370521_at	Vps33b	3.2931E-03	-1.22
1389448_at	Sep-04	3.4118E-03	-1.25
1389638_at	Fam160a2	3.4489E-03	-1.27
1378229_at	---	3.4807E-03	-1.58
1384337_x_at	Cln3	3.4918E-03	-1.32
1369928_at	Acta1	3.5154E-03	-1.13
1390275_at	LOC681714 /// L(	3.6127E-03	-1.25
1397180_at	Styxl1	3.6300E-03	-1.50
1379800_at	Znf532	3.6322E-03	-1.06
1399159_a_at	Vamp3	3.6813E-03	-1.16
1370074_at	Baiap2	3.7370E-03	-1.23
1374427_at	---	3.7951E-03	-1.76
1378935_at	Sox17	3.8738E-03	-1.10
1368652_at	Casp9	3.9835E-03	-1.25
1388870_at	Msi2	3.9939E-03	-1.10
1373112_at	RGD1306873	4.0187E-03	-1.34
1376210_at	Foxo3	4.1195E-03	-1.21
1380573_a_at	RGD1564722	4.2105E-03	-1.16
1378309_at	Usp20	4.3025E-03	-1.18
1372475_at	Pink1	4.3098E-03	-1.12
1370880_at	Rnh1	4.4715E-03	-1.08
1373223_at	---	4.5647E-03	-1.39
1370121_at	Add1	4.6460E-03	-1.52
1376577_at	---	4.7661E-03	-2.41
1373923_at	Rdh10	4.7868E-03	-1.22
1372780_at	Tmem53	4.8282E-03	-1.34
1374543_at	---	4.9533E-03	-1.27
1374417_at	Nrbp2	4.9726E-03	-1.35
1396048_at	Cachd1	5.0125E-03	-2.01

1377417_at	LOC363328	5.0215E-03	-1.19
1372314_at	Wdr26	5.0394E-03	-1.03
1375494_a_at	Nlgn3	5.1622E-03	-1.24
1384132_at	Cadm1	5.1932E-03	-1.68
1386943_at	Pllp	5.2841E-03	-1.39
1392625_at	---	5.3194E-03	-1.11
1375249_at	Eif2c1	5.3202E-03	-1.30
1393966_at	Pbx1	5.3641E-03	-1.32
1378102_at	Pcnxl2	5.3699E-03	-1.13
1395791_at	RGD1306404	5.4122E-03	-1.51
1382134_at	Olfml1	5.4310E-03	-1.72
1387615_at	St8sia2	5.4691E-03	-1.63
1390523_at	---	5.5261E-03	-1.40
1383205_at	Dact2	5.5763E-03	-2.14
1370850_at	Scn3b	5.6901E-03	-1.19
1373995_at	Abcg1	5.6916E-03	-1.46
1371775_at	---	5.7469E-03	-1.84
1389213_at	Vash1	5.7515E-03	-1.27
1386543_at	Fbxl8	5.7653E-03	-1.24
1380108_at	Wbp1	5.8082E-03	-1.18
1378034_at	Mterfd3	5.8661E-03	-1.20
1389251_at	Nudt7	5.8812E-03	-1.49
1382285_at	Naga	5.9616E-03	-1.54
1382985_at	---	5.9719E-03	-1.18
1394609_at	Ablim2	5.9951E-03	-1.55
1368153_a_at	Nelf	6.0995E-03	-1.30
1387057_at	Slc7a8	6.1328E-03	-1.84
1373884_at	---	6.2011E-03	-1.12
1398484_at	RGD1308221	6.2244E-03	-1.38
1378860_at	---	6.2367E-03	-1.53
1370063_at	Nr2f2	6.2987E-03	-1.53
1373376_at	---	6.3847E-03	-1.14
1368138_at	Mapt	6.4286E-03	-1.21
1386635_at	---	6.4362E-03	-1.22
1381027_at	---	6.5668E-03	-1.66
1390226_at	RGD1562552	6.6045E-03	-2.65
1390038_at	Adcy5	6.6203E-03	-1.15
1392174_at	Chst12	6.7034E-03	-1.43
1370574_a_at	Sirpa	6.7091E-03	-1.85
1394028_at	Dusp10	6.7520E-03	-1.82
1387304_at	Uts2r	6.8325E-03	-1.17

1385087_at	---	7.0465E-03	-1.21
1371629_at	Cxxc5	7.1695E-03	-1.11
1377067_at	---	7.1904E-03	-1.48
1396255_at	Kif16b	7.2392E-03	-1.63
1382579_at	Tox3	7.2694E-03	-1.49
1390536_at	---	7.2791E-03	-1.39
1371613_at	Elmo2	7.3103E-03	-1.25
1375084_at	Serinc2	7.3129E-03	-1.22
1395269_s_at	Gabrd	7.3512E-03	-1.25
1380515_at	Bbs7	7.5237E-03	-1.41
1382670_at	Larp1	7.5316E-03	-1.31
1381719_at	---	7.5693E-03	-1.06
1368276_at	Syp	7.5993E-03	-1.42
1378224_at	---	7.6486E-03	-1.18
1371595_at	---	7.6772E-03	-3.00
1367835_at	Pcsk1n	7.6849E-03	-1.56
1389142_at	Sqrdl	7.6966E-03	-1.17
1372772_at	Nfat5	7.8306E-03	-1.14
1398973_at	Tm9sf3	7.8517E-03	-1.37
1398650_at	LOC683844 /// L(	7.9098E-03	-1.10
1374089_at	---	7.9177E-03	-1.69
1388083_a_at	Casp9	8.0131E-03	-1.40
1382118_at	Apeg3	8.0223E-03	-2.15
1368879_a_at	Gnao1	8.1140E-03	-1.47
1368064_a_at	Ddc	8.1325E-03	-1.43
1382490_at	---	8.1597E-03	-1.29
1398616_at	Ap1s2	8.2811E-03	-1.37
1398741_at	---	8.3059E-03	-1.22
1375307_at	Cbx6	8.3373E-03	-1.43
1371004_at	Sort1	8.3644E-03	-1.24
1387375_at	Khk	8.5667E-03	-1.68
1378563_at	RGD1308722	8.6289E-03	-1.29
1373292_at	Orai3 /// RGD131	8.6666E-03	-1.11
1372767_at	Fam168b /// LOC	8.6713E-03	-1.12
1389289_at	Ewsr1	8.7248E-03	-1.23
1397267_at	Rimbp2	8.8046E-03	-1.69
1372836_at	---	8.8202E-03	-1.24
1386685_at	Sacs	9.0108E-03	-1.22
1393994_at	Rpl13a	9.1433E-03	-1.20
1394270_at	Mks1	9.2386E-03	-1.13
1370992_a_at	Fga	9.3568E-03	-1.17

1395453_at	RGD1560871	9.4056E-03	-1.21
1382240_at	---	9.4777E-03	-1.34
1373291_at	---	9.4809E-03	-1.53
1371251_at	Galt	9.4950E-03	-1.28
1383262_at	---	9.4984E-03	-1.19
1372724_at	Grina	9.5000E-03	-1.39
1368778_at	Slc6a6	9.6015E-03	-1.46
1386324_at	---	9.6411E-03	-1.09
1389313_at	---	9.8233E-03	-1.23
1369909_s_at	Tmem150	9.8588E-03	-1.82
1371020_at	Rimbp2	9.8671E-03	-1.65
1398332_at	Ppm1f	9.9393E-03	-1.20
1388781_at	---	1.0059E-02	-1.09
1388572_at	LOC301128	1.0135E-02	-1.27
1369941_at	Dap	1.0259E-02	-1.34
1384056_at	Fgfr3	1.0370E-02	-1.64
1378954_at	---	1.0404E-02	-1.11
1370590_at	Gpsm1	1.0408E-02	-1.27
1372953_at	Ncald	1.0421E-02	-1.83
1376297_at	Arrdc1	1.0514E-02	-1.30
1371587_at	Map2k1ip1	1.0657E-02	-1.10
1373789_at	---	1.0665E-02	-1.15
1398330_at	Stxbp1	1.0724E-02	-1.32
1377091_at	Prrt1	1.0731E-02	-1.53
1369757_at	Itpkb	1.0736E-02	-2.60
1370658_a_at	St18	1.0870E-02	-1.65
1370544_at	Eml2	1.0888E-02	-1.72
1372773_at	Npdc1	1.0953E-02	-1.29
1391820_at	RGD1309285	1.0986E-02	-1.29
1388624_at	RGD1561113	1.0996E-02	-1.36
1373503_at	Lrch4	1.1050E-02	-1.34
1383727_at	---	1.1057E-02	-1.12
1399052_at	Tollip	1.1354E-02	-1.27
1391598_at	---	1.1374E-02	-1.43
1372544_at	---	1.1500E-02	-1.12
1382928_at	Pdzd3	1.1501E-02	-1.55
1367726_at	Thra	1.1502E-02	-1.27
1372132_at	Cndp2	1.1593E-02	-1.29
1378685_at	LOC689836	1.1676E-02	-1.28
1376219_at	LOC685152	1.1724E-02	-1.32
1387577_at	Neurod2	1.1751E-02	-1.45

1387155_at	Pcsk2	1.1982E-02	-1.99
1395599_at	---	1.1991E-02	-1.23
1388837_at	Slc44a2	1.1993E-02	-1.40
1397788_at	---	1.2124E-02	-1.51
1368099_at	Clasp2	1.2136E-02	-1.14
1372382_at	---	1.2200E-02	-1.16
1368869_at	Akap12	1.2240E-02	-2.22
1374019_at	RGD1564379	1.2320E-02	-1.31
1380203_at	Znf512b	1.2431E-02	-1.13
1374388_at	Efhd2	1.2463E-02	-1.33
1378691_at	---	1.2535E-02	-1.39
1388415_at	Ctnnd1	1.2605E-02	-1.35
1389206_at	---	1.2636E-02	-1.45
1395309_at	---	1.2686E-02	-1.07
1383994_at	Syt3	1.2727E-02	-1.40
1374165_at	RGD1565457	1.2906E-02	-1.16
1381063_at	---	1.2941E-02	-1.34
1388245_a_at	Nrg1	1.2977E-02	-1.08
1388006_at	Muc13	1.3135E-02	-1.82
1381243_at	---	1.3177E-02	-1.40
1391587_at	---	1.3212E-02	-1.42
1393965_at	---	1.3314E-02	-2.03
1390930_at	---	1.3329E-02	-1.22
1388633_at	RGD1309313	1.3418E-02	-1.38
1385667_x_at	---	1.3422E-02	-1.09
1372258_at	LOC100192313	1.3451E-02	-1.21
1395600_at	Ccdc117	1.3572E-02	-1.36
1383159_at	Tom1l2	1.3626E-02	-1.31
1369515_s_at	Insrr	1.3748E-02	-1.63
1390555_at	Socs5	1.3756E-02	-1.32
1381964_at	RGD1563047	1.3793E-02	-1.24
1388121_at	Aplp2	1.3914E-02	-1.48
1386379_at	---	1.4036E-02	-1.16
1373095_at	RGD1307597	1.4042E-02	-1.12
1370474_at	Thrb	1.4061E-02	-1.63
1397701_at	---	1.4084E-02	-1.27
1368176_at	Rara	1.4176E-02	-1.47
1385773_at	---	1.4348E-02	-1.53
1382325_at	Gcat	1.4582E-02	-2.22
1398335_at	Ttyh3	1.4588E-02	-1.28
1372050_at	Glt25d1	1.4618E-02	-1.16

1377094_at	---	1.4721E-02	-1.15
1373971_at	Lpcat4	1.4895E-02	-1.63
1392888_at	Gpc4	1.4914E-02	-1.31
1385018_at	Dsg1b	1.5009E-02	-1.13
1371618_s_at	Tubb3	1.5110E-02	-1.30
1391177_at	Txlna	1.5199E-02	-1.37
1368251_at	Jak3	1.5256E-02	-1.13
1378384_at	Ints6	1.5365E-02	-1.94
1393688_at	LOC685048 /// LC	1.5412E-02	-1.17
1368206_at	Acot8	1.5436E-02	-1.08
1380053_at	Tm9sf3	1.5486E-02	-1.62
1390172_at	Dhtkd1	1.5709E-02	-2.30
1367982_at	Alas1	1.5718E-02	-1.14
1369332_a_at	Rims1	1.5941E-02	-1.71
1373637_at	---	1.5965E-02	-1.69
1371916_at	Sepx1	1.5982E-02	-1.22
1372087_at	Iah1	1.6050E-02	-1.36
1399019_at	Abhd4	1.6122E-02	-1.10
1388561_at	---	1.6156E-02	-1.24
1393947_at	Slc25a15	1.6160E-02	-1.11
1379737_a_at	Rbm39	1.6200E-02	-1.11
1378524_at	Rnf19a	1.6247E-02	-1.32
1394605_at	RGD1566149	1.6286E-02	-1.13
1375199_at	---	1.6329E-02	-1.32
1372626_at	Tpd52l1	1.6333E-02	-1.21
1385826_at	---	1.6374E-02	-1.14
1370362_at	Ptprn	1.6377E-02	-1.22
1393182_at	Cxcl3	1.6521E-02	-1.12
1380963_at	---	1.6591E-02	-1.42
1374573_at	Dync2li1	1.6647E-02	-1.04
1387426_at	Slc25a21	1.6659E-02	-1.12
1393937_at	---	1.6719E-02	-2.00
1380523_at	Fbxo15	1.6751E-02	-1.09
1374933_at	Mcam	1.6798E-02	-1.05
1384238_at	Ttyh2	1.6889E-02	-1.11
1387310_at	Atp2c2	1.7016E-02	-1.87
1388848_at	RGD1308350	1.7054E-02	-1.28
1388705_at	Selm	1.7069E-02	-1.36
1396325_at	Zfp819	1.7072E-02	-1.19
1382963_at	Abcb10	1.7295E-02	-1.40
1379970_at	Epha4	1.7302E-02	-1.25

1386239_at	Dmrta1	1.7350E-02	-4.55
1388768_at	Sgsm2	1.7362E-02	-1.15
1397627_at	Diaph1	1.7362E-02	-1.35
1393893_at	Morn5	1.7395E-02	-1.19
1379156_at	---	1.7407E-02	-1.25
1397209_at	RGD1565712	1.7473E-02	-1.43
1391757_at	---	1.7483E-02	-2.70
1377839_at	Agap3	1.7526E-02	-1.12
1370225_at	Cited4	1.7571E-02	-1.29
1377214_a_at	RGD1303117	1.7594E-02	-1.26
1373586_at	---	1.7675E-02	-1.17
1374996_at	Nploc4	1.7695E-02	-1.15
1391618_at	Kifap3	1.7734E-02	-1.17
1376257_at	Znf629	1.7799E-02	-1.40
1369681_at	Isl1	1.7800E-02	-1.11
1369212_s_at	Epb4.1l1	1.7818E-02	-1.17
1396257_at	---	1.7961E-02	-1.24
1374441_at	Spryd4	1.7968E-02	-1.18
1373985_at	Pnmal2	1.8020E-02	-1.70
1397607_at	RGD1310862	1.8021E-02	-1.24
1372989_at	Zdhhc14	1.8203E-02	-1.96
1384589_at	Spata2L	1.8264E-02	-1.29
1392822_at	Nf1	1.8278E-02	-1.14
1377996_at	---	1.8324E-02	-1.11
1372024_at	---	1.8357E-02	-1.22
1395145_at	Rgl1	1.8509E-02	-1.51
1371994_at	Arhgap1	1.8614E-02	-1.22
1394961_at	---	1.8642E-02	-2.14
1369632_a_at	Abcc8	1.8667E-02	-1.20
1396179_at	LOC684425	1.8672E-02	-1.36
1398984_at	Tm2d2	1.8845E-02	-1.17
1368477_at	Atp2a3	1.8890E-02	-1.63
1372193_at	Impact	1.8891E-02	-1.35
1383695_at	Vipr1	1.8943E-02	-1.64
1387720_at	Clstn2	1.9018E-02	-1.35
1389554_at	---	1.9037E-02	-1.69
1387783_a_at	Acaa1 /// RGD15	1.9057E-02	-1.48
1370693_a_at	Cnp	1.9125E-02	-1.73
1369327_at	Pdzd2	1.9135E-02	-1.46
1377744_at	---	1.9202E-02	-1.65
1395068_at	RGD1566029	1.9222E-02	-1.66

1377929_at	---	1.9301E-02	-1.21
1382674_a_at	RGD1310027	1.9356E-02	-1.44
1390887_at	RGD1309995	1.9379E-02	-1.14
1396928_at	---	1.9425E-02	-1.22
1377720_x_at	---	1.9454E-02	-1.33
1368992_a_at	Sfrs5	1.9544E-02	-1.31
1390529_at	Cd83	1.9561E-02	-1.33
1398994_at	Tpst2	1.9606E-02	-1.11
1375806_at	Tnfaip2	1.9678E-02	-1.19
1377726_at	Trim25	1.9697E-02	-1.16
1376697_at	Chst12	1.9707E-02	-1.37
1378236_at	Lrrc16a	1.9844E-02	-1.75
1369159_at	Ar	1.9939E-02	-1.25
1370431_at	Syn2	2.0009E-02	-1.17
1376584_at	Ppm1l	2.0019E-02	-1.24
1375182_at	---	2.0080E-02	-1.34
1382254_at	---	2.0091E-02	-1.14
1372390_at	Apeg3	2.0175E-02	-1.40
1368513_at	Enpep	2.0177E-02	-2.09
1392692_at	---	2.0188E-02	-1.26
1390103_at	Phf2	2.0196E-02	-1.20
1399167_a_at	Gab1	2.0247E-02	-1.27
1367538_at	---	2.0263E-02	-1.13
1397622_at	---	2.0270E-02	-1.22
1367748_at	Arf5	2.0364E-02	-1.62
1369942_at	Actn4	2.0438E-02	-1.34
1369325_at	Lyst	2.0513E-02	-1.27
1378140_at	Arl11	2.0704E-02	-1.24
1389789_at	Mxd1	2.0735E-02	-1.24
1382563_at	RGD1560818	2.0741E-02	-1.56
1373894_at	Rab31	2.0929E-02	-1.14
1392996_at	Cpeb1	2.0930E-02	-1.53
1369999_a_at	Nnat	2.1042E-02	-1.21
1371343_at	Srpr	2.1146E-02	-1.04
1381692_x_at	---	2.1156E-02	-1.26
1370232_at	Ivd	2.1198E-02	-1.68
1387015_at	Pfn2	2.1236E-02	-1.17
1371799_at	Gaa	2.1242E-02	-1.34
1373843_at	---	2.1271E-02	-1.86
1395735_at	RGD1310271	2.1352E-02	-1.19
1382940_at	Glb1l2	2.1357E-02	-1.84

1387154_at	Npy	2.1520E-02	-1.58
1393404_at	Rtn4rl1	2.1582E-02	-1.53
1389635_at	---	2.1598E-02	-1.35
1374905_at	---	2.1638E-02	-1.18
1377719_a_at	---	2.1764E-02	-1.33
1376583_at	RGD1304884	2.1833E-02	-1.17
1383074_at	Man2a1	2.1909E-02	-1.64
1394475_at	---	2.1960E-02	-1.20
1369112_at	Chrm3	2.1962E-02	-1.46
1369330_at	Unc13a	2.2373E-02	-1.27
1373579_at	Rara	2.2459E-02	-1.65
1389889_at	RGD1306404	2.2486E-02	-1.68
1368482_at	Bcl2a1d	2.2573E-02	-1.06
1368387_at	Bdh1	2.2752E-02	-1.33
1373533_at	---	2.2763E-02	-1.91
1380282_at	Cmip	2.2905E-02	-1.80
1384390_at	LOC681740	2.2932E-02	-1.16
1396298_at	---	2.2996E-02	-1.16
1368603_at	Add2	2.3004E-02	-1.14
1391117_at	Pnmal2	2.3078E-02	-1.44
1376673_at	---	2.3279E-02	-1.29
1376347_at	Jarid1b	2.3327E-02	-1.21
1397521_at	Ilvbl	2.3352E-02	-2.00
1392561_at	Atp5c1	2.3425E-02	-1.20
1378457_at	---	2.3426E-02	-1.53
1390725_at	LOC691396	2.3486E-02	-1.35
1386945_a_at	Prkab1	2.3503E-02	-1.11
1380811_at	---	2.3626E-02	-1.48
1387983_at	Thrb	2.3799E-02	-1.63
1380526_at	Gcdh	2.3823E-02	-1.44
1375648_at	RGD1307966	2.3878E-02	-1.52
1388579_at	Slc25a42	2.3891E-02	-1.35
1369069_at	Akap1	2.3901E-02	-2.09
1392811_at	Usp40	2.4009E-02	-1.28
1387190_at	Dgka	2.4093E-02	-1.32
1393495_at	---	2.4099E-02	-1.40
1374428_at	Kif3b	2.4113E-02	-1.22
1395271_at	Rbm27	2.4191E-02	-1.48
1376863_at	---	2.4214E-02	-1.06
1387288_at	Neurod1	2.4312E-02	-1.46
1370451_a_at	Cacna1c	2.4374E-02	-1.27

1398615_at	Garnl4	2.4383E-02	-1.53
1375657_at	---	2.4421E-02	-1.54
1390340_a_at	Eif4g1	2.4564E-02	-1.66
1390838_at	Satb2	2.4635E-02	-1.53
1370046_at	Polg	2.4649E-02	-1.49
1372719_at	---	2.4853E-02	-1.52
1397515_at	B4galnt4	2.4865E-02	-2.40
1389914_at	RGD1563533	2.5059E-02	-1.24
1396252_at	---	2.5075E-02	-1.39
1386960_at	Slc37a4	2.5121E-02	-1.30
1380459_at	Btbd14a	2.5255E-02	-1.30
1390871_at	Tcerg1	2.5323E-02	-1.56
1374845_at	---	2.5326E-02	-1.20
1371112_at	Ret	2.5341E-02	-2.05
1370159_at	Smarcd2	2.5367E-02	-1.31
1373306_at	Slc25a44	2.5385E-02	-1.28
1378239_at	Gaa	2.5402E-02	-2.02
1386851_a_at	Ppt2	2.5413E-02	-1.30
1367966_at	Dpp3 /// LOC678	2.5447E-02	-1.29
1388418_at	Bcl9	2.5732E-02	-1.56
1385034_at	Lhx9	2.5815E-02	-1.16
1368985_at	Grin2a	2.5905E-02	-1.14
1367548_at	Suds3	2.5916E-02	-1.32
1368246_at	Ap3m2	2.5970E-02	-1.23
1391113_at	Rhbdl3	2.6007E-02	-1.53
1376225_at	---	2.6009E-02	-1.71
1387409_x_at	Nlgn3	2.6010E-02	-1.19
1373018_at	Spryd3	2.6152E-02	-1.41
1377140_at	Fam171a1 /// LO	2.6208E-02	-1.42
1380983_at	---	2.6217E-02	-1.19
1379581_at	Mapk1ip1l	2.6323E-02	-1.41
1378338_at	Ammecr1l	2.6334E-02	-1.70
1390362_at	---	2.6340E-02	-1.12
1387997_at	Hcn4	2.6392E-02	-1.38
1389392_at	Clcn6	2.6417E-02	-1.48
1368868_at	Akap12	2.6472E-02	-2.57
1385449_s_at	Vps37b	2.6500E-02	-1.61
1385420_at	Dvl3	2.6505E-02	-1.40
1383723_at	Specc1l	2.6619E-02	-1.18
1398231_at	---	2.6689E-02	-2.02
1372587_at	Emcn	2.6698E-02	-1.12

1386224_at	---	2.6769E-02	-1.18
1368215_at	Tpp1	2.6810E-02	-1.46
1376355_at	FAM120C	2.6830E-02	-1.23
1387708_at	Adra2a	2.6840E-02	-1.38
1387698_at	Kcnj11	2.6938E-02	-1.62
1383640_at	---	2.6962E-02	-1.09
1372782_a_at	Ampd2	2.7009E-02	-1.26
1391434_at	Rab3a	2.7030E-02	-1.23
1379254_at	Tmem183a	2.7049E-02	-1.11
1369066_at	Madd	2.7115E-02	-1.62
1381399_at	LOC690840 /// R	2.7136E-02	-1.19
1385897_at	---	2.7213E-02	-1.20
1382090_at	---	2.7227E-02	-1.24
1380189_at	---	2.7308E-02	-1.44
1383328_x_at	Pdcd4	2.7447E-02	-1.50
1375965_at	Ube2k	2.7457E-02	-1.31
1394954_at	Slc31a2	2.7466E-02	-2.70
1386076_at	---	2.7507E-02	-1.23
1370989_at	Ret	2.7544E-02	-2.07
1398755_at	Atp6v0c	2.7587E-02	-1.31
1397813_at	---	2.7614E-02	-1.73
1387609_at	Car5a	2.7616E-02	-1.11
1387023_at	Gstm7	2.7690E-02	-1.15
1369222_at	Kcnt1	2.7705E-02	-1.22
1375011_at	---	2.7706E-02	-1.59
1390076_at	---	2.7741E-02	-1.19
1397741_at	Ankrd52	2.7817E-02	-1.16
1370114_a_at	Pik3r1	2.7824E-02	-1.37
1389833_at	---	2.7938E-02	-1.34
1374085_at	Mxd4	2.7956E-02	-1.32
1376713_at	Mta1	2.7972E-02	-1.22
1372958_at	Otub1	2.7979E-02	-1.11
1390837_at	---	2.8055E-02	-1.33
1396389_at	LOC687758	2.8062E-02	-1.09
1385306_at	LOC687346	2.8075E-02	-1.26
1381543_at	Slc38a7	2.8151E-02	-1.85
1395610_at	Tspan33	2.8254E-02	-1.57
1383298_at	RGD1310444	2.8266E-02	-1.55
1396238_at	Galnt14	2.8431E-02	-1.56
1394979_at	---	2.8525E-02	-1.53
1376907_at	---	2.8548E-02	-1.32

1395356_at	LOC683953 /// Pı	2.8566E-02	-1.84
1368340_at	Ipmk	2.8587E-02	-1.28
1391714_at	Plag1	2.8596E-02	-1.11
1379913_at	---	2.8636E-02	-1.86
1390192_at	Slc27a3	2.8651E-02	-1.13
1396541_at	Jmjd1c	2.8673E-02	-2.01
1395019_at	Usp38	2.8678E-02	-1.31
1390426_at	Notch1	2.8748E-02	-1.47
1369771_at	Irs1	2.8795E-02	-1.12
1371141_at	Slc25a27	2.8824E-02	-1.20
1398512_at	Nfx1	2.8827E-02	-1.22
1387797_at	Rab7a	2.8918E-02	-1.20
1397207_at	Otud4	2.8961E-02	-1.78
1383969_at	---	2.8967E-02	-1.19
1388058_at	Taf6	2.8993E-02	-1.17
1386793_at	Zfp61	2.9042E-02	-1.29
1369827_at	Clstn3	2.9084E-02	-1.65
1371578_at	Prkaca	2.9214E-02	-1.41
1386333_at	---	2.9269E-02	-1.25
1388788_at	Gcdh	2.9381E-02	-1.27
1382561_at	---	2.9397E-02	-2.08
1378591_at	---	2.9429E-02	-1.16
1370964_at	Ass1	2.9522E-02	-2.09
1367602_at	Cited2	2.9574E-02	-1.82
1370750_a_at	Il1r1	2.9610E-02	-3.23
1386870_at	Glul	2.9619E-02	-1.56
1399118_at	RGD1564778	2.9737E-02	-1.23
1390191_at	---	2.9751E-02	-1.13
1373757_at	Trafd1	2.9763E-02	-1.22
1381775_at	---	2.9770E-02	-1.42
1372858_at	---	2.9872E-02	-2.19
1374202_at	---	2.9900E-02	-1.31
1396079_at	RGD1311429	3.0095E-02	-1.53
1377707_at	Slc35b4	3.0142E-02	-1.45
1372624_at	Ano6	3.0193E-02	-1.48
1398975_at	Aamp	3.0195E-02	-1.19
1397496_at	RGD1560620	3.0230E-02	-1.27
1379700_at	Hs1bp3	3.0281E-02	-1.57
1387517_at	Syt13	3.0284E-02	-2.12
1369592_at	Wbp2	3.0322E-02	-1.85
1380625_at	---	3.0378E-02	-1.28

1379544_at	---	3.0401E-02	-1.09
1383379_at	LOC684112	3.0449E-02	-1.39
1375662_at	---	3.0467E-02	-1.55
1368385_a_at	Grb2	3.0476E-02	-1.35
1369728_at	LOC684887	3.0489E-02	-1.16
1377068_at	Tpst1	3.0605E-02	-1.23
1372163_at	---	3.0644E-02	-1.41
1375540_at	Cdkn2aipnl	3.0657E-02	-1.40
1369635_at	Sord	3.0673E-02	-1.42
1382339_a_at	---	3.0724E-02	-1.60
1372041_at	---	3.0769E-02	-1.54
1385226_at	Kctd11	3.0780E-02	-1.13
1379794_at	Gzmb	3.0802E-02	-1.08
1376678_at	Apbb2	3.0861E-02	-1.37
1369203_at	Wif1	3.0905E-02	-1.70
1395582_at	---	3.0937E-02	-1.16
1391643_at	---	3.0942E-02	-1.15
1371522_at	Bahd1	3.1019E-02	-1.14
1389232_at	Wdr5b	3.1032E-02	-1.17
1391901_at	---	3.1090E-02	-2.21
1369518_at	Pik3r3	3.1234E-02	-2.37
1371485_at	---	3.1239E-02	-1.92
1370785_s_at	Tomm20	3.1271E-02	-1.19
1375992_at	RGD1564114	3.1333E-02	-1.53
1386998_at	Aldoc	3.1343E-02	-1.67
1379190_at	Oxct2a	3.1430E-02	-1.09
1391013_at	Pcdh8	3.1433E-02	-1.34
1394849_at	Zbtb20	3.1446E-02	-1.29
1385912_at	Dusp15	3.1449E-02	-1.22
1393822_at	Tmcc3	3.1461E-02	-1.59
1395251_at	---	3.1463E-02	-1.72
1384844_at	RGD1564005	3.1481E-02	-1.88
1394404_at	Strn4	3.1678E-02	-1.32
1397826_at	Epb4.9	3.1696E-02	-1.63
1377863_at	Jarid1b	3.1729E-02	-1.28
1394464_at	LOC690830 /// R	3.1801E-02	-1.09
1376455_at	Mfsd3	3.1839E-02	-1.13
1373558_at	---	3.1869E-02	-1.18
1370606_at	P2ry1	3.1906E-02	-2.26
1370210_at	Tmem158	3.1941E-02	-1.39
1397887_at	---	3.1982E-02	-1.40

1383934_at	---	3.2025E-02	-1.55
1372610_at	P4ha2	3.2065E-02	-1.31
1388157_at	Marcks	3.2096E-02	-1.74
1386867_at	Brp44l	3.2100E-02	-1.28
1373598_at	Ubn1	3.2142E-02	-1.32
1368103_at	Abcg1	3.2178E-02	-1.36
1370527_a_at	Csnk1d	3.2179E-02	-1.61
1369574_at	Tas2r13	3.2181E-02	-1.05
1380260_at	Barx1	3.2250E-02	-1.12
1389423_at	Ddr2	3.2251E-02	-1.28
1381850_at	Ppp1r12a	3.2286E-02	-2.07
1370993_at	Lamc1	3.2361E-02	-1.36
1395401_at	Dos	3.2371E-02	-1.50
1382279_at	Rhobtb2	3.2412E-02	-1.35
1397425_at	Rabgap1	3.2470E-02	-1.17
1373512_at	Ilvbl	3.2471E-02	-1.62
1387585_at	Slc29a2	3.2554E-02	-1.25
1390463_at	---	3.2571E-02	-1.29
1389043_at	Adck4	3.2590E-02	-1.24
1370701_at	Gabrq	3.2601E-02	-1.26
1379629_at	---	3.2621E-02	-1.27
1382099_at	Vps26a	3.2632E-02	-1.74
1368876_a_at	Nrxn2	3.2811E-02	-1.32
1387415_a_at	Stxbp5	3.2821E-02	-1.25
1397164_at	---	3.2920E-02	-4.24
1369758_at	Gpam	3.2931E-02	-1.25
1375733_at	---	3.2994E-02	-1.33
1375338_at	Rab10	3.3022E-02	-1.29
1373104_at	Specc1l	3.3046E-02	-1.13
1391693_at	Atp8b1	3.3057E-02	-1.27
1379822_at	Sfrs18	3.3171E-02	-1.26
1387387_at	Hpca	3.3189E-02	-1.69
1371530_at	Krt8	3.3203E-02	-1.25
1387830_at	Crp	3.3280E-02	-2.52
1381860_at	---	3.3427E-02	-1.11
1395337_at	Tjp1	3.3440E-02	-1.46
1378221_at	Srfbp1	3.3455E-02	-1.16
1368081_at	Abca2	3.3468E-02	-1.15
1398631_at	Gtpbp6	3.3498E-02	-1.10
1380820_at	---	3.3538E-02	-1.15
1387899_at	Crmp1	3.3543E-02	-1.20

1369969_at	Parp1	3.3614E-02	-1.16
1377498_at	Ampd2	3.3621E-02	-1.50
1384927_at	Tubb4	3.3624E-02	-1.22
1376012_at	RGD1564419	3.3625E-02	-1.14
1398406_at	---	3.3694E-02	-1.32
1379904_at	Trappc6a	3.3701E-02	-1.32
1376593_at	---	3.3790E-02	-1.41
1392632_at	Ttc9	3.3797E-02	-1.41
1373657_at	Slc31a2	3.3913E-02	-1.99
1368388_at	Maf	3.4051E-02	-1.10
1369699_at	Glp1r	3.4152E-02	-1.41
1392645_at	---	3.4214E-02	-1.69
1375484_at	---	3.4230E-02	-1.28
1373520_at	---	3.4256E-02	-1.30
1395346_at	Aamp	3.4359E-02	-1.55
1368008_at	Prom1	3.4392E-02	-1.30
1397571_at	Casc3	3.4394E-02	-1.46
1387859_at	Nfs1	3.4473E-02	-1.41
1396356_at	Elk4	3.4512E-02	-1.24
1371702_at	Tspan7	3.4529E-02	-1.37
1389370_at	Phf1	3.4531E-02	-1.18
1386604_at	Mpp3	3.4660E-02	-1.43
1373304_at	Coasy	3.4720E-02	-1.29
1398982_at	Rab1b	3.4752E-02	-1.14
1381799_at	RGD1562037	3.4772E-02	-1.96
1376208_at	---	3.4781E-02	-1.75
1397819_at	Prr14	3.4798E-02	-1.33
1368213_at	Por	3.4812E-02	-1.57
1371961_at	Pld3	3.4847E-02	-1.19
1371558_at	Nisch	3.4865E-02	-1.14
1382196_at	RGD1307394	3.4880E-02	-1.80
1383084_at	---	3.4912E-02	-1.46
1379408_at	RGD1305020	3.4941E-02	-1.29
1392993_at	Fam13b1	3.4966E-02	-1.53
1386338_at	---	3.5040E-02	-1.22
1379274_at	---	3.5058E-02	-1.34
1397744_at	---	3.5119E-02	-1.32
1394797_at	---	3.5166E-02	-1.27
1369093_at	Reln	3.5176E-02	-1.16
1395441_at	Pcbp4	3.5216E-02	-1.53
1389969_at	Tomm40	3.5224E-02	-1.21

1377165_at	---	3.5272E-02	-1.26
1389286_at	Glrx5	3.5313E-02	-1.19
1371307_at	Rplp1	3.5373E-02	-1.15
1370209_at	Klf9	3.5379E-02	-1.31
1386512_at	Zfp91	3.5535E-02	-1.10
1375177_at	Klf13	3.5616E-02	-1.14
1391752_at	---	3.5717E-02	-1.30
1376978_at	---	3.5771E-02	-1.24
1395556_at	LOC503175	3.5820E-02	-2.04
1368690_a_at	Grm4	3.5973E-02	-1.21
1370819_at	Csnk1d	3.5977E-02	-1.42
1384943_at	RGD1563888	3.6056E-02	-1.23
1390845_at	---	3.6094E-02	-1.31
1387025_at	Dync1i1	3.6095E-02	-1.26
1397526_at	Gcdh	3.6096E-02	-2.01
1396217_at	---	3.6244E-02	-1.38
1374566_at	---	3.6274E-02	-1.15
1383573_at	Tshz1	3.6298E-02	-1.49
1380285_at	Chrd	3.6355E-02	-1.48
1392988_at	Nsdhl	3.6427E-02	-1.22
1369081_at	Neu1	3.6493E-02	-1.75
1381672_at	Ldlrap1	3.6500E-02	-1.29
1384621_at	---	3.6510E-02	-1.24
1395454_at	Ostm1	3.6550E-02	-1.24
1376153_at	---	3.6569E-02	-1.42
1394721_at	Baz2a	3.6584E-02	-1.20
1392581_at	Ncald	3.6605E-02	-2.46
1385777_at	LOC681647	3.6611E-02	-1.64
1367736_at	Rraga	3.6617E-02	-1.34
1383330_at	---	3.6652E-02	-1.24
1380202_at	Znf821	3.6653E-02	-1.24
1377838_at	RGD1562218	3.6666E-02	-1.06
1399138_at	Wdr42a	3.6674E-02	-1.42
1389177_at	Perp	3.6679E-02	-1.45
1369919_at	Tef	3.6702E-02	-1.71
1381656_at	---	3.6705E-02	-1.16
1367955_at	Rab4b	3.6839E-02	-1.25
1382270_at	---	3.6957E-02	-1.43
1367725_at	Pim3	3.7022E-02	-1.26
1382931_at	---	3.7078E-02	-1.15
1370573_at	Sardh	3.7113E-02	-1.73

1371165_a_at	Atp2a3	3.7193E-02	-2.70
1393629_at	Hlx	3.7197E-02	-1.38
1378544_at	Rbbp6	3.7229E-02	-1.39
1373109_at	Map2k7	3.7233E-02	-1.33
1396322_at	---	3.7304E-02	-1.12
1393016_s_at	Sec3l1	3.7318E-02	-1.50
1385610_at	Sephs1	3.7403E-02	-1.08
1376736_at	LOC688990	3.7473E-02	-2.30
1397396_at	RGD1308297	3.7515E-02	-1.13
1384187_at	Ap1s2	3.7530E-02	-1.51
1394926_at	---	3.7630E-02	-1.12
1375197_at	Uqcr	3.7647E-02	-1.16
1391835_at	---	3.7655E-02	-1.26
1368105_at	Tspan2	3.7708E-02	-1.22
1389305_at	Anxa4	3.7831E-02	-1.26
1372358_at	LOC686590	3.7901E-02	-1.07
1392451_at	---	3.8018E-02	-1.71
1371854_at	---	3.8060E-02	-1.34
1377240_at	---	3.8095E-02	-1.62
1369539_at	St3gal3	3.8166E-02	-2.05
1375656_at	RGD1306873	3.8181E-02	-1.53
1387169_at	Tle3	3.8232E-02	-1.16
1375013_at	---	3.8263E-02	-1.06
1368874_a_at	Mafg	3.8273E-02	-1.54
1379167_at	---	3.8302E-02	-1.19
1387268_at	Polr1b	3.8336E-02	-1.33
1371063_at	Sh3gl2	3.8434E-02	-1.29
1390998_at	Glis1	3.8550E-02	-1.27
1387421_at	Kcnip3	3.8597E-02	-2.38
1388997_at	Arf3	3.8646E-02	-1.36
1393361_at	RGD1310922	3.8649E-02	-1.33
1393486_at	Cyp46a1	3.8693E-02	-1.18
1387095_at	Gnaz	3.8697E-02	-2.50
1392852_at	Smoc1	3.8826E-02	-1.62
1372526_at	Flcn	3.8848E-02	-1.53
1374501_at	---	3.8849E-02	-1.18
1379953_at	Pptc7	3.8891E-02	-1.40
1370248_at	Fxyd6	3.8974E-02	-1.48
1374419_at	---	3.9044E-02	-1.29
1390810_at	---	3.9155E-02	-1.07
1371029_at	Pkd1	3.9316E-02	-1.31

1373534_at	Sfrs18	3.9317E-02	-1.21
1368847_at	Rab10	3.9440E-02	-1.51
1367569_at	Rpsa	3.9452E-02	-1.05
1390552_at	Magi1	3.9486E-02	-1.15
1392883_at	RGD1305269	3.9501E-02	-1.65
1374976_a_at	Soat1	3.9553E-02	-2.19
1369384_at	Gria4	3.9561E-02	-1.22
1389047_at	Bag2	3.9582E-02	-1.20
1374625_at	Hes6	3.9617E-02	-1.17
1380314_at	RGD1563072	3.9650E-02	-1.56
1382946_a_at	Rab1b /// Rab1b-	3.9707E-02	-1.46
1369255_at	Il1r1	3.9857E-02	-2.39
1388249_at	Rapgef1	3.9888E-02	-2.18
1391601_at	Lrrn2	3.9951E-02	-1.35
1377550_at	Slc25a23	3.9953E-02	-1.30
1390639_at	---	4.0010E-02	-1.14
1377849_at	Vcpip1	4.0010E-02	-1.35
1392841_at	---	4.0101E-02	-1.21
1379169_at	Slc25a23	4.0129E-02	-1.54
1385692_at	Slc10a4	4.0137E-02	-1.06
1392882_at	RGD1305110	4.0184E-02	-1.68
1367960_at	Arl4a	4.0197E-02	-1.36
1368514_at	Maob	4.0309E-02	-1.83
1393296_at	Ate1	4.0314E-02	-1.37
1369804_a_at	Csnk1e	4.0316E-02	-1.77
1375856_at	---	4.0343E-02	-1.24
1394802_at	Syt7	4.0344E-02	-1.98
1393438_at	Zfp316	4.0380E-02	-1.29
1384163_at	---	4.0435E-02	-1.31
1387489_at	Extl3	4.0622E-02	-1.66
1390431_at	---	4.0685E-02	-1.33
1389479_at	Klf3	4.0716E-02	-1.37
1375516_at	Ndufc2	4.0794E-02	-1.09
1376216_at	LOC367812 /// Sp	4.0802E-02	-1.36
1395914_at	Csnk1d	4.0952E-02	-1.62
1379899_at	---	4.0977E-02	-1.09
1382496_at	Hnf4a	4.0997E-02	-1.64
1378817_at	Itsn1	4.1025E-02	-1.11
1396886_at	---	4.1056E-02	-1.25
1371958_at	Pabpn1	4.1071E-02	-1.42
1388347_at	Ly6e	4.1102E-02	-1.22

1368343_at	Kcnh2	4.1178E-02	-1.20
1392021_at	---	4.1190E-02	-1.50
1394664_at	---	4.1300E-02	-1.22
1379627_at	Tm9sf3	4.1315E-02	-1.80
1368411_a_at	Map2	4.1369E-02	-1.80
1395308_at	Tmem127	4.1404E-02	-1.33
1376794_at	---	4.1421E-02	-1.22
1382850_at	Syn2	4.1474E-02	-1.93
1388811_at	---	4.1518E-02	-1.37
1384188_at	---	4.1519E-02	-2.16
1393630_at	LOC688272	4.1619E-02	-1.60
1378355_a_at	Slc24a4	4.1626E-02	-1.29
1368573_at	Kpnb1	4.1635E-02	-1.37
1397711_at	---	4.1664E-02	-1.07
1379160_at	---	4.1713E-02	-2.45
1372383_at	Gpsm1	4.1856E-02	-1.23
1377461_at	LOC680485	4.1888E-02	-1.81
1384161_at	Csnk1e	4.1912E-02	-1.32
1384974_at	LOC691024	4.1961E-02	-2.24
1387412_at	Pip4k2a	4.2078E-02	-1.23
1383501_at	---	4.2123E-02	-1.28
1368137_at	Mapt	4.2231E-02	-1.18
1390655_at	---	4.2262E-02	-3.27
1381173_at	---	4.2267E-02	-1.12
1397052_at	Rbbp6	4.2268E-02	-1.41
1389801_at	Fgfr4	4.2524E-02	-1.31
1393355_at	---	4.2524E-02	-1.09
1375288_at	Gcn1l1	4.2534E-02	-1.22
1388993_at	Rnf34	4.2541E-02	-1.51
1382712_at	---	4.2583E-02	-1.68
1374724_at	---	4.2596E-02	-2.01
1378000_at	---	4.2598E-02	-1.28
1375978_at	Fcho1	4.2666E-02	-1.80
1383435_at	Scn3b	4.2725E-02	-1.13
1369316_s_at	Snap29	4.2785E-02	-1.78
1378745_at	Per3	4.2843E-02	-1.19
1392905_at	Gng2	4.2843E-02	-1.12
1393610_at	Fam76a	4.2995E-02	-1.41
1399155_at	LOC296637	4.3105E-02	-1.91
1382269_at	Cnnm2	4.3118E-02	-1.60
1387874_at	Dbp	4.3138E-02	-1.53

1371093_at	Znf291	4.3158E-02	-1.32
1391480_at	---	4.3170E-02	-1.26
1395706_at	Lingo1	4.3180E-02	-2.21
1384963_at	---	4.3197E-02	-1.30
1395332_at	Tbx2	4.3198E-02	-1.06
1384708_at	---	4.3198E-02	-1.35
1369548_at	Gtf2a1	4.3289E-02	-1.23
1372378_at	Dis3l2	4.3341E-02	-1.15
1373624_at	---	4.3343E-02	-1.59
1393661_at	---	4.3395E-02	-1.42
1398810_at	Pdap1	4.3407E-02	-1.23
1387130_at	Slc40a1	4.3408E-02	-1.48
1394946_at	rCG_42077 /// R(	4.3468E-02	-1.17
1392701_at	Gmppb	4.3489E-02	-1.11
1368821_at	Fstl1	4.3501E-02	-1.17
1394572_at	---	4.3521E-02	-1.09
1384209_at	---	4.3562E-02	-1.22
1397965_at	Porcn	4.3587E-02	-1.13
1368850_at	Csnk1g3	4.3602E-02	-1.35
1388070_a_at	Akap1	4.3645E-02	-2.07
1378094_at	---	4.3651E-02	-1.25
1370882_at	Hla-dmb	4.3654E-02	-1.08
1383863_at	Lmo2	4.3699E-02	-1.32
1383587_at	RGD1565496	4.3710E-02	-1.96
1383161_a_at	---	4.3818E-02	-1.33
1371066_at	Snrk	4.3851E-02	-1.43
1376134_at	RGD1307789	4.3865E-02	-1.30
1369249_at	Ankh	4.3955E-02	-1.56
1397747_at	Xpo7	4.4012E-02	-1.28
1380532_at	LOC689593	4.4041E-02	-1.17
1369096_at	Epha7	4.4085E-02	-1.20
1390317_at	RGD1561849	4.4135E-02	-1.21
1368327_at	Slc12a9	4.4157E-02	-1.23
1367651_at	Ctsd	4.4266E-02	-1.47
1385120_at	Pof1b	4.4273E-02	-1.20
1388020_a_at	Pde1c	4.4274E-02	-1.15
1380623_at	Taz	4.4366E-02	-1.33
1386876_at	Adcy6	4.4398E-02	-1.60
1393593_at	Mar-06	4.4414E-02	-1.54
1396137_at	Zer1	4.4489E-02	-1.60
1394635_at	---	4.4585E-02	-1.27

1380772_at	---	4.4586E-02	-1.15
1381984_at	---	4.4656E-02	-1.13
1377425_at	Zbtb2	4.4725E-02	-1.18
1393604_at	Gpn3	4.4736E-02	-1.44
1383848_at	Adrb1	4.4746E-02	-1.11
1385232_at	Trim62	4.4750E-02	-1.65
1372057_at	Nrbp	4.4852E-02	-1.23
1372263_at	LOC296637	4.4888E-02	-1.42
1395991_at	---	4.4997E-02	-1.31
1396435_at	---	4.5002E-02	-1.18
1391992_at	---	4.5013E-02	-1.14
1368641_at	Wnt4	4.5099E-02	-1.20
1367647_at	Serpina1	4.5109E-02	-2.39
1392547_at	MGC105649	4.5124E-02	-1.58
1381539_at	---	4.5197E-02	-1.19
1386917_at	Pc	4.5272E-02	-1.10
1398797_at	Hnrnpk	4.5402E-02	-1.12
1369400_a_at	Pfkfb2	4.5475E-02	-1.75
1372766_at	Cpne5	4.5565E-02	-1.31
1383834_at	Agap3	4.5576E-02	-1.27
1383035_at	Ffar2	4.5696E-02	-1.63
1375310_at	Impact	4.5704E-02	-1.20
1396187_at	Ddx21	4.5707E-02	-1.83
1397960_at	Armcx3	4.5731E-02	-2.20
1389851_at	Zfp36l2	4.5732E-02	-1.71
1375207_at	Scarf2	4.5834E-02	-1.24
1383755_at	Fkbp15	4.5872E-02	-1.90
1375608_at	---	4.5881E-02	-1.18
1369002_at	Soat1	4.5896E-02	-3.12
1390446_at	---	4.5922E-02	-1.28
1387406_at	Uhmk1	4.5984E-02	-1.51
1378967_at	Hnrpdl	4.6010E-02	-1.26
1395435_at	---	4.6043E-02	-1.78
1373691_at	---	4.6110E-02	-1.19
1369099_at	Slc30a1	4.6149E-02	-2.77
1371696_at	Gpr56	4.6214E-02	-1.60
1385724_at	LOC682874	4.6296E-02	-1.17
1367767_at	Hmgcl	4.6315E-02	-1.31
1374081_at	Casc4	4.6327E-02	-1.32
1387888_at	Rps9	4.6351E-02	-1.12
1378517_at	---	4.6352E-02	-1.33

1381162_at	Sep-10	4.6435E-02	-1.15
1387340_at	Rtn3	4.6488E-02	-1.16
1397391_at	---	4.6513E-02	-1.17
1398300_at	Atp1b3	4.6614E-02	-1.17
1376637_at	---	4.6696E-02	-1.97
1385187_at	Glt25d1	4.6723E-02	-1.45
1383049_at	Klhl8	4.6733E-02	-1.45
1386643_at	Fem1b	4.6927E-02	-1.36
1383557_at	---	4.6961E-02	-1.31
1397215_at	---	4.7043E-02	-1.07
1387038_at	Ccs	4.7053E-02	-1.20
1376952_at	---	4.7123E-02	-1.55
1398466_at	Calr4	4.7197E-02	-1.15
1373898_at	Pan3	4.7208E-02	-1.14
1397282_at	---	4.7311E-02	-1.89
1371640_at	Znf706	4.7406E-02	-1.36
1376868_at	Cobll1	4.7408E-02	-1.56
1379860_at	---	4.7548E-02	-1.45
1388365_at	Atp6v0d1	4.7650E-02	-1.14
1386035_at	---	4.7658E-02	-1.55
1389725_at	Tm7sf2	4.7708E-02	-2.06
1379673_at	Uap1l1	4.7728E-02	-2.24
1397047_at	---	4.7759E-02	-1.48
1377366_at	---	4.7816E-02	-1.33
1372882_at	Dnlz	4.7870E-02	-1.23
1393976_at	Slbp	4.7986E-02	-1.15
1389393_at	Fam108c1	4.8000E-02	-1.35
1377785_at	Dhx40	4.8007E-02	-1.30
1378927_at	---	4.8068E-02	-1.21
1379069_at	LOC100192314	4.8117E-02	-1.14
1387285_at	Atp2b2	4.8128E-02	-1.82
1375517_at	Trp53inp2	4.8224E-02	-1.30
1384069_at	Lrp11	4.8225E-02	-1.31
1379325_at	Mkrn1	4.8267E-02	-1.41
1393833_at	Angel1	4.8276E-02	-1.11
1383631_at	RGD1311563	4.8435E-02	-1.17
1383619_at	Mtmr4	4.8436E-02	-1.48
1387847_at	Pik3cb	4.8444E-02	-1.36
1384856_at	RGD1563946	4.8460E-02	-1.41
1392378_at	Klhdc3	4.8507E-02	-1.33
1378293_at	Trim26	4.8556E-02	-1.14

1389100_at	Epm2aip1	4.8572E-02	-1.98
1391094_at	---	4.8580E-02	-1.41
1373173_at	---	4.8630E-02	-1.23
1369656_at	Pcyt1a	4.8755E-02	-1.64
1377235_a_at	Ppp2r5b	4.8811E-02	-1.15
1397742_at	---	4.8880E-02	-1.12
1391573_at	Tnfrsf21	4.8883E-02	-1.43
1371059_at	Prkar2a	4.8954E-02	-1.90
1371733_at	Arl8a	4.8957E-02	-1.46
1382199_at	---	4.8966E-02	-1.51
1385176_at	---	4.9062E-02	-1.32
1390819_at	Tef	4.9156E-02	-1.30
1384832_at	Ppfia1	4.9160E-02	-1.65
1374401_at	Snx2	4.9199E-02	-1.18
1370478_at	Myo16	4.9199E-02	-1.68
1398478_at	Mlh3	4.9256E-02	-1.31
1395367_at	Rnf215	4.9269E-02	-1.52
1377887_at	---	4.9273E-02	-1.14
1372210_at	Mospd3	4.9300E-02	-1.13
1375187_at	---	4.9321E-02	-1.12
1377877_at	Nlp	4.9324E-02	-1.31
1388799_at	Klhl7	4.9390E-02	-1.59
1385926_at	---	4.9453E-02	-1.26
1370650_s_at	Bdkrb2 /// RGD1	4.9456E-02	-1.18
1371957_at	Imp4	4.9563E-02	-1.07
1382867_at	---	4.9587E-02	-1.16
1393586_at	Nope	4.9598E-02	-1.03
1383580_at	---	4.9605E-02	-1.40
1369782_a_at	Kcnj11	4.9610E-02	-1.88
1369688_s_at	Ptk2b	4.9614E-02	-1.69
1378751_at	Mapk4	4.9627E-02	-1.94
1385212_at	---	4.9683E-02	-1.28
1377050_at	---	4.9753E-02	-1.19
1378228_at	LOC683953 /// P	4.9770E-02	-1.40
1388261_at	Drp2	4.9777E-02	-1.16
1380570_at	---	4.9800E-02	-1.29
1370597_at	Stx17	4.9863E-02	-1.61
1394566_at	RGD1305500	4.9935E-02	-1.25
1388409_at	Zc3h7b	4.9950E-02	-1.27
1378194_a_at	Rufy3	4.9977E-02	-1.70
1388426_at	Srebf1	4.9996E-02	-1.34
