# Supplementary material for: Glucolipotoxicity initiates pancreatic β-cell death through TNFR5/CD40-mediated STAT1 and NF-κB activation
Source: Cell Death Dis. 2016 Aug 11;7(8):e2329–. doi: 10.1038/cddis.2016.203 (PMC5108311; doi:10.1038/cddis.2016.203)
Supplement: Supplementary Appendix Table 1 [file cddis2016203x2.pdf]

	**Affymetrix**	**PCR**
IFNγ induced GTPase	1.80 ($p<0.05$)	2.65 ($p<0.001$)
IFNγ inducible protein 30	1.78 ($p<0.05$)	2.08 ($p<0.001$)
IFNγ receptor 1	1.26 ($p<0.05$)	2.28 ($p<0.001$)
IFN stimulated exonuclease 20	1.41 ($p<0.001$)	2.01 ($p<0.001$)
IFN-induced protein 44	1.37 ($p<0.05$)	3.04 ($p<0.001$)
IFN, α-inducible protein	1.38 ($p<0.05$)	2.52 ($p<0.001$)
