# Supplementary material for: Glucolipotoxicity initiates pancreatic β-cell death through TNFR5/CD40-mediated STAT1 and NF-κB activation
Source: Cell Death Dis. 2016 Aug 11;7(8):e2329–. doi: 10.1038/cddis.2016.203 (PMC5108311; doi:10.1038/cddis.2016.203)
Supplement: Supplementary Information [file cddis2016203x4.doc]

**Appendix Table 1**

Significant differentially expressed genes in INS-1 β-cells following exposure to a glucolipotoxic environment using Partek ® software, based on a p value < 0.05 with a fold change greater or less than 2.

**Appendix Table 2**

Expression of interferon-associated genes was determined using Affymetrix array data and independent qRT-PCR analysis. Data is shown from three independent experiments.

**Appendix Figure 1**

Metacore (http://genego.com) integrated knowledge database legend describing details of network objects and interactions.
